# Evidence that the rab5 effector APPL1 mediates APP-βCTF-induced dysfunction of endosomes in Down syndrome and Alzheimer's disease

**DOI:** 10.1038/mp.2015.97

**Published:** 2015-07-21

**Authors:** S Kim, Y Sato, P S Mohan, C Peterhoff, A Pensalfini, A Rigoglioso, Y Jiang, R A Nixon

**Affiliations:** 1 Cellular and Molecular Biology Training Program, New York University School of Medicine, New York, NY, USA; 2 Center for Dementia Research, Nathan S Kline Institute for Psychiatric Research, Orangeburg, NY, USA; 3 Department of Psychiatry, New York University School of Medicine, New York, NY, USA; 4 Department of Cell Biology, New York University School of Medicine, New York, NY, USA

## Abstract

β-Amyloid precursor protein (APP) and its cleaved products are strongly implicated in Alzheimer's disease (AD). Endosomes are highly active APP processing sites, and endosome anomalies associated with upregulated expression of early endosomal regulator, rab5, are the earliest known disease-specific neuronal response in AD. Here, we show that the rab5 effector APPL1 (adaptor protein containing pleckstrin homology domain, phosphotyrosine binding domain and leucine zipper motif) mediates rab5 overactivation in Down syndrome (DS) and AD, which is caused by elevated levels of the β-cleaved carboxy-terminal fragment of APP (βCTF). βCTF recruits APPL1 to rab5 endosomes, where it stabilizes active GTP-rab5, leading to pathologically accelerated endocytosis, endosome swelling and selectively impaired axonal transport of rab5 endosomes. In DS fibroblasts, APPL1 knockdown corrects these endosomal anomalies. βCTF levels are also elevated in AD brain, which is accompanied by abnormally high recruitment of APPL1 to rab5 endosomes as seen in DS fibroblasts. These studies indicate that persistent rab5 overactivation through βCTF–APPL1 interactions constitutes a novel APP-dependent pathogenic pathway in AD.

## Introduction

β-Amyloid precursor protein (APP) and its cleaved product, amyloid-β peptide (Aβ), are strongly implicated in Alzheimer's disease (AD) via β-amyloid toxicity, although disease pathogenesis is increasingly considered multifactorial,^1, 2^ possibly involving additional APP metabolites.^1, 3, 4^ Endosomes are highly active APP processing sites and genes that influence endocytosis are over-represented as AD risk factors.^4, 5, 6^ Endosome anomalies associated with upregulated expression of rab5 and other endocytosis-related genes are the earliest known disease-specific neuronal response in AD.^7, 8^ They develop early in Down syndrome (DS, Trisomy 21),^8^ a cause of early-onset AD linked to an extra copy of APP, wherein APP-dependent endosome abnormalities are associated with late endosome anomalies^9^ and defective endosomal signaling,^10^ leading to cholinergic neurodegeneration in DS brains.^11^ Similar endosome dysfunction is seen in neurons generated from induced pluripotent stem cells from individuals with familial and sporadic AD patients^4^ and DS fibroblasts.^3^ In particular, endosomal abnormalities found in DS cells are caused by the β-cleaved carboxy-terminal fragment of APP (βCTF).^3^ Endocytosis is particularly important in neurons for receptor trafficking, neurotrophin signaling and neurotransmission.^12^ It is also critical for regulating nuclear signaling via endosome-mediated interactions of APPL1 (adaptor protein containing pleckstrin homology domain, phosphotyrosine binding (PTB) domain and leucine zipper motif), a binding partner and effector of rab5.^13, 14^ APPL1 is localized mainly in early endosomal membrane.^15^ There are also populations of APPL1 vesicles distinct from rab5-positive endosomes, although their identity has not been determined.^13^ APPL1 on rab5-positive endosomes translocates from endosomal membranes to the nucleus where it regulates chromatin structure and gene expression.^13^ It also mediates several signaling processes, including the nuclear factor-κB (NF-κB) and insulin pathways,^16^ the Akt pathway via phosphoinositides^17^ and epidermal growth factor receptor signaling.^18^ The small GTPase, rab5, regulates these processes and controls diverse signaling and cell functions of early endosomes.^19^ Abnormal activation of rab5 is implicated in AD- and DS-related endosome dysfunction;^8, 9, 11^ however, the mechanism underlying pathological rab5 activation in AD is unknown.

Although it has been shown that βCTF can promote rab5-mediated endosomal pathology in DS fibroblasts,^3^ it is not known to interact directly with rab5. Although rab5 activation-induced clathrin-dependent APP endocytosis has been suggested to participate in βCTF and amyloid-β production through a rab5-dependent pathway,^20^ it is not clear how rab5 overactivation contributes to disease onset and progression. Here, we show that APPL1 mediates rab5 activation caused by elevated levels of βCTF in DS and AD. By binding the PTB domain of APPL1, βCTF recruits APPL1 to endosomes, where it stabilizes active GTP-rab5 and increases rab5 activity on endosomes, leading to pathologically accelerated endocytosis, followed by AD-like endosome swelling and selectively impaired axonal transport of endosomes in neurons. In fibroblasts from individuals with DS, small interfering RNA (siRNA) silencing of APPL1 corrects known endocytic anomalies^3^ and reverses elevated nuclear translocation of p65/RelA, an indication of activated NF-κB signaling,^21^ which is known to be mediated by APPL1/rab5 endosomes.^16^ Finally, we show, for the first time, that βCTF levels are elevated in AD despite normal APP levels, which is accompanied by abnormally high recruitment of APPL1 to rab5 endosomes in AD brain, similar to that seen in cells from individuals with DS. These studies indicate that persistent rab5 overactivation through βCTF–APPL1 interactions constitutes a novel βCTF-dependent and Aβ-independent, pathogenic pathway contributing to the development of AD and DS.

## Materials and methods

### Cell culture and transfection

Embryonic mouse cortical neurons from E17 to E18 pregnant C57BL/6J females were cultured as described previously.^22^ All animal studies were performed with an approved protocol from the Nathan Kline Institute Institutional Animal Care and Use Committee.

### Statistics

Results are presented as mean±s.e.m. Unless otherwise noted, statistical significance was determined by unpaired two-tailed Student's *t*-test for two-sample *t*-test and one-way analysis of variance (ANOVA), followed by Tukey's test to determine statistical significance for multiple comparisons. A *P*-value <0.05 was considered statistically significant.

Details of the Materials and methods are given in Supplementary Materials and Methods.

## Results

### βCTF activates rab5 and induces endosomal enlargement

**Figure 1:**
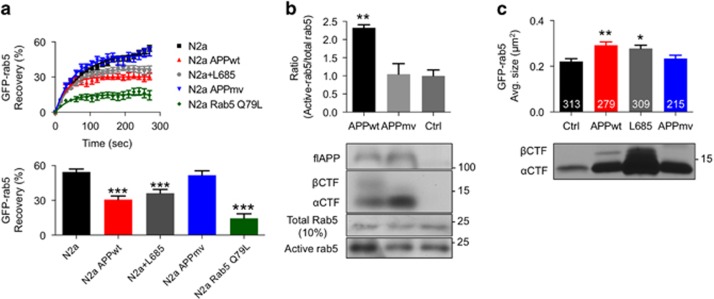
β-Cleaved carboxy-terminal fragment of APP (βCTF) activates rab5 on endosomes and increases endosome size. (**a**) Rab5 activation, reflected by the rate of fluorescence recovery after photobleaching (FRAP), is measured as the rate at which photobleached GTP-bound rab5 (activated rab5) on individual endosomes is replaced by fluorescent cytosolic GDP-rab5 (inactive rab5). The rate of FRAP for endosomal GFP-rab5 is significantly decreased when βCTF levels are increased by overexpressing wild-type APP (APPwt) in N2a cells (N2a APPwt) or blocking βCTF cleavage with a 10 μm γ-secretase inhibitor L685 458 (N2a+L685), as compared with control cells expressing βCTF at endogenous levels (N2a) or transfected with an APP mutant construct unable to be cleaved to βCTF (N2a APPmv). The FRAP rate after transfecting a dominant-active mutant GFP-rab5 Q79L^25^ as a positive control is extremely reduced, indicating persistent rab5 activation. The summary graph reflects by the percent of average fluorescence recovery at 270 s after photobleaching in each condition (*n*=20 endosomes, one endosome per cell, total 20 cells, mean±s.e.m., one-way analysis of variance (ANOVA), Tukey's test, ***P<0.001). (**b**) βCTF activates rab5 in human embryonic kidney 293 (HEK293) cells. APPwt overexpression increases the levels of active rab5 (GTP-bound rab5) detected with a GTP-rab5-specific antibody (first lane), whereas APPmv mutant has no effect (second lane), as compared with untransfected control (ctrl) cells (third lane). A bar graph presents mean immunoreactive GTP-rab5 signal±s.e.m. for three separate immunoblot experiments (representative blot shown) (**P<0.01, one-way ANOVA, Tukey's test). Ten percent of cell lysates is used as total rab5. (**c**) βCTF induces endosomal enlargement in N2a cells. Cross-sectional area of rab5-positive endosomes is increased by APPwt overexpression or 10μM L685 458 (lanes 2 and 3), which raise αCTF and βCTF levels compared with those in ctrl cells (lane 1). APPmv mutant expression elevates αCTF levels but not βCTF levels (lane 4) and does not enlarge endosomes. Each graph bar indicates the number of measured endosomes from 20 cells (*P<0.05 and **P<0.01, respectively, mean±s.e.m., one-way ANOVA, Tukey's test).

To characterize APP influences on endocytosis, we first analyzed N2a mouse neuroblastoma cells overexpressing wild-type human APP stably (N2aAPP). N2aAPP cells internalized horseradish peroxidase (HRP) twofold more rapidly compared with N2a cells (Supplementary Figure 1a), consistent with evidences of accelerated endocytosis in DS fibroblasts measured by transferrin, HRP and epidermal growth factor uptake.^3, 9^ We documented rab5 activation by three assays. First, we detected 50% higher levels of membrane-bound rab5 (GTP-loaded active form) in N2aAPP cells compared with N2a cells using crude membrane fractionation analysis (Supplementary Figure 1b). Second, we measured rates of fluorescence recovery after photobleaching (FRAP) on GFP-rab5 endosomes to reveal rab5 flux through the GDP-GTP cycle, which requires dissociation of bleached GFP-rab5 from endosomal membranes and insertion of unbleached GFP-rab5 from the surrounding cytosol.^23^ The rate of rab5 exchange provides an indication of its activation state.^23^ As expected, endosomes in N2a cells expressing GFP-rab5 Q79L, a dominant-active mutant unable to hydrolyze GTP,^24^ showed a very diminished rate of recovery of fluorescence, indicating the constitutively activated state of the rab5 mutant (Figure 1a). Overexpression of wild-type human APP (APPwt) in N2a cells also significantly decreased the rate of FRAP on endosomes when compared with that in control N2a cells, indicating greater rab5 activation (Figure 1a). In a third approach, we directly measured levels of active rab5 by selectively immunoprecipitating this form with an antibody specific for GTP-rab5. This immunoblot analysis confirmed increased GTP-rab5 levels in human embryonic kidney 293 (HEK293) cells expressing APPwt compared with control cells, indicating that APPwt overexpression is sufficient to increase active rab5 levels (Figure 1b). Consistent with actions of activated rab5 in promoting endosome fusion,^25^ GFP-rab5-positive endosomes in N2a cells overexpressing APPwt, on average, were significantly larger compared with those in control N2a cells based on morphometric analyses, providing an independent functional evidence of rab5 activation (Figure 1c). Taken together, these data show that APPwt overexpression activates rab5 on endosomes, upregulating endocytosis and endosomal fusion,^25^ which promotes endosome enlargement, as seen in early AD and DS.^8^

Because one particular APP cleavage product, βCTF, is known to produce endosomal pathology in DS cells,^3, 4^ we tested if βCTF generation was sufficient to induce rab5 activation by using a mutant APP M596V (APPmv) that is unable to generate βCTF.^26^ Expressing APPmv had no effect on endosome FRAP in N2a cells (Figure 1a), active rab5 levels in HEK293 cells (Figure 1b) or endosome size in N2a cells (Figure 1c), whereas a γ-secretase inhibitor (L685 458) significantly increased the levels of βCTF by blocking its γ-cleavage to Aβ^3^ (Figure 1c), slowed GTP-rab5 fluorescence recovery (Figure 1a) and enlarged endosomes (Figure 1c). Consistent with previous findings in DS fibroblasts,^3^ endosomal enlargement was not dependent on αCTF levels that were higher in APPmv-overexpressed cells compared with that in control N2a cells (Figure 1c), confirming that rab5 activation is mediated specifically by βCTF. Similar to the pattern in AD brain,^27^ endosomal enlargement was disproportionately greater in the endosome population >0.5μm^2^ (Supplementary Figure 1c). Thus, these studies show that APP-dependent rab5 activation requires β-cleavage of APP but not α- or γ-cleavages, as we previously showed in DS fibroblasts.^3^

### βCTF selectively binds via its YENPTY domain to the PTB domain of APPL1

**Figure 2:**
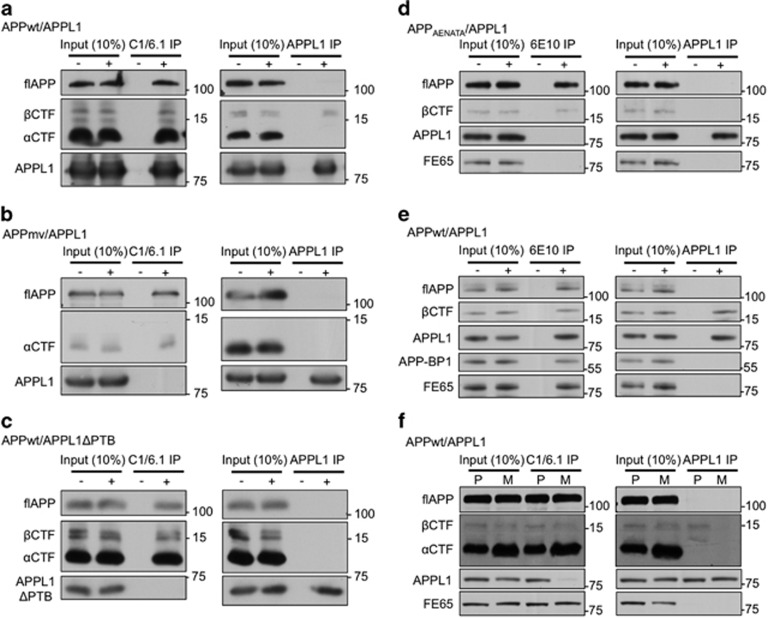
β-Cleaved carboxy-terminal fragment of APP (βCTF) generated on endosomes binds via its YENPTY domain to the PTB domain of APPL1 (adaptor protein containing pleckstrin homology domain, phosphotyrosine binding domain and leucine zipper motif). (**a**) βCTF interacts with APPL1: immunoblot analysis shows co-immunoprecipitation (co-IP) of βCTF and APPL1 from cell lysates of human embryonic kidney 293 (HEK293) cells overexpressing wild-type human APP (APPwt) and APPL1 using antibodies against APPL1 or against APP (APP C1/6.1 antibody is raised against amino-acid residues 676–695 of human APP695 and recognizes full-length APP (flAPP), αCTF and βCTF). (**b**) APPL1 interaction with APP requires β-secretase cleavage: APPL1 does not co-IP with a β-cleavage-incompetent mutant APP (APPmv) from lysates of HEK293 cells overexpressing both proteins. (**c**) βCTF–APPL1 interaction requires the PTB domain of APPL1: βCTF does not co-IP with a mutant APPL1 (APPL1ΔPTB) lacking the PTB domain when this mutant and APPwt are overexpressed in HEK293 cells. (**d**) APPL1 interacts via the YENPTY domain of βCTF: APPL1 does not co-IP with an APP mutant containing a YENPTY domain variant (APP_AENATA_) from lysates of HEK293 cells overexpressing both proteins. (**e**) FE65 and APP-BP1 are immunoprecipitated with APP using 6E10 antibody, raised against the amino acids 1–17 of human Aβ, detects human flAPP, βCTF and Aβ but not with APPL1 from lysates of HEK293 cells overexpressing APPwt and APPL1. (**f**) APPL1 selectively interacts with endocytosed βCTF: in N2a cells overexpressing APPwt and APPL1, APPL1 does not co-IP with βCTF when βCTF generation on endosomes is prevented by blocking endocytosis with MiTMAB (M), a dynamin inhibitor. Treatment with promyristic acid (P), an inactive form of MiTMAB, a negative control for endocytic blockade, does not prevent APPL1wt co-IP with βCTF. In all co-IP experiments, 10% of total cell lysates is used as an input standard. (+) and (−) indicates the presence and absence of the antibody used in IP, respectively.

As APP is not known to interact directly with rab5, we sought a protein mediator of βCTF-dependent rab5 activation, capitalizing on the knowledge that APP interacts with proteins containing the PTB domain.^28^ We considered APPL1 a good candidate because it contains the PTB domain and also has other domains mediating selective binding to active GTP-bound rab5, which then stabilizes rab5 in its activated GTP state on endosomal membranes and recruits additional rab5.^13, 29^ Moreover, perisomatic APPL1 distribution is altered in AD brain.^30^ Based on double-immunolabeling analyses of APPL1 and GFP-rab5, APPwt overexpression in N2a cells indeed increased APPL1 recruitment to rab5-positive endosomes (Supplementary Figure 2a). To identify the interaction between APP and APPL1, we carried out a series of co-immunoprecipitation (co-IP) analyses in transfected HEK293 cells. In lysates from cells overexpressing APPwt and APPL1wt, an antibody (C1/6.1) against the extreme C-terminal end of APP pulled down APPL1 along with full-length APP (flAPP), βCTF and αCTF, whereas a specific APPL1 antibody precipitated APPL1 only with βCTF and not flAPP and αCTF (Figure 2a). Similar analysis using cells overexpressing the APPmv mutant and APPL1 yielded no APPL1-βCTF co-IP (Figure 2b), indicating that APPL1 selectively interacts with βCTF. Similarly, an overexpressed mutant APPL1 (APPL1ΔPTB) lacking the PTB domain did not co-IP with βCTF using either antibody (Figure 2c), suggesting that the PTB domain of APPL1 is required for βCTF binding.

Because some proteins that contain the PTB domain have been shown to interact with the YENPTY motif of APP,^28, 31^ we tested a mutant APP (APP_AENATA_) that contains AENATA instead of YENPTY^32^ to see if this domain is important for the interaction between APPL1 and βCTF. Because C1/6.1 does not recognize this construct owing to the mutation, we used 6E10 antibody against amino acids 1–17 of human Aβ, which recognizes APP_AENATA_. In cells overexpressing both mutant APP and APPL1, the APP_AENATA_ mutant did not co-IP APPL1 using 6E10, and APPL1 antibody no longer co-immunoprecipitated βCTF (Figure 2d), suggesting that the YENPTY motif of βCTF mediated its binding to APPL1. FE65, an adaptor protein containing the PTB domain and known to interact with APP via the YENPTY motif,^33^ was also unable to bind to APP_AENATA_ (Figures 2d and e), confirming that the mutation disrupts the interaction with the PTB domain.

APP-BP1, an NEDD8-activating enzyme E1 regulatory subunit, is known to interact with the carboxy terminal of APP and also with rab5 to promote endosomal enlargement.^10, 34^ It is possible, therefore, that APP-BP1 could mediate the interaction between APPL1 and βCTF. Using APPL1 and 6E10 antibodies, we found that, whereas APP-BP1 was able to bind to APP, it did not co-IP with APPL1 in lysates of cells overexpressing both proteins (Figure 2e), suggesting that APP-BP1 does not mediate APPL1–βCTF interaction.

flAPP and αCTF are mainly localized on the plasma membrane, whereas βCTF is mainly generated on endosomes.^35^ Blocking endocytosis should therefore diminish the association of βCTF with an endosomal adaptor protein, such as APPL1. To test this idea, we treated N2a cells overexpressing APPwt/APPL1wt with MiTMAB, a cell-permeable inhibitor for dynamin I and II to block endocytosis, or promyristic acid, an inactive form of MiTMAB, as a negative control and performed co-IP using C1/6.1 or APPL1 antibody (Figure 2f). MiTMAB reduced βCTF levels (Figure 2f) as shown previously^36^ and increased αCTFs (Figure 2f) as expected given that this cleavage is considered to take place at the plasma membrane.^36, 37^ MiTMAB significantly decreased the interaction of APPL1 with βCTF (Figure 2f and Supplementary Figure 2b), whereas FE65, which is known to interact with APP in the plasma membrane,^38^ was still able to bind to APP (Figure 2f). Taken together, these analyses show that APPL1 specifically binds to internalized βCTF through the interaction between PTB and YENPTY domains.

### APPL1 knockdown reverses βCTF-induced rab5 activation and endosomal enlargement

**Figure 3:**
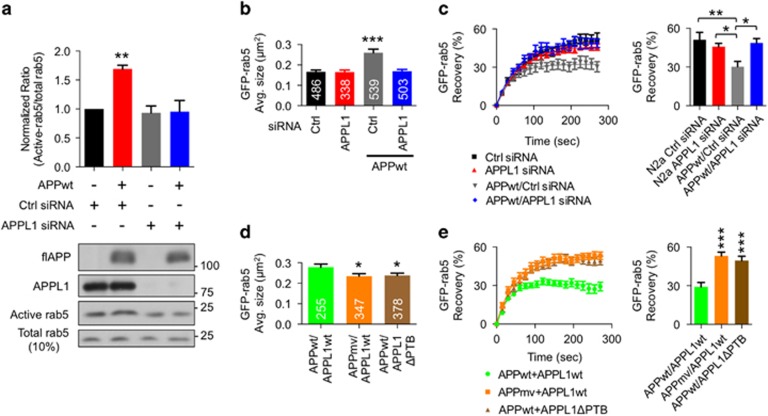
APPL1 (adaptor protein containing pleckstrin homology domain, phosphotyrosine binding domain and leucine zipper motif) mediates β-cleaved carboxy-terminal fragment of APP (βCTF)-induced endosomal enlargement and rab5 activation. (**a**) APPL1 small interfering RNA (siRNA) knockdown blocks wild-type human APP (APPwt)-mediated Rab5 activation: APPwt overexpression in human embryonic kidney 293 (HEK293) cells significantly elevates the ratio of activated (GTP-bound) rab5 to total rab5 relative to the ratio in cells lacking APPwt overexpression (lane 2 vs lane 1) (**P<0.01, one-way analysis of variance (ANOVA), Tukey's test). siRNA knockdown of APPL1 prevents rab5 activation in the presence of APPwt overexpression (fourth lane) but has no effect on rab5 activation when APP is expressed at endogenous levels (third lane). The bar graph depicts mean±s.e.m. for four separate experiments (one representative immunoblot shown). (**b**) APPL1 mediates APPwt-induced rab5 enlargement. Increases in average size (cross-sectional area) of GFP-rab5 endosomes induced by APPwt overexpression in N2a cells are reversed with APPL1 siRNA treatment but not with scrambled control siRNA (ctrl) (***P<0.001, one-way ANOVA, Tukey's test). APPL1 siRNA does not affect endosomal size in cells expressing APP at endogenous levels. Each graph bar indicates the number of measured endosomes from 20 cells and shows values as mean±s.e.m. Ten percent of total cell lysates is used as a standard. (**c**) APPL1 mediates APPwt-induced rab5 activation on endosomes: APPL1 siRNA, but not control siRNA, blocks the slowed fluorescence recovery after photobleaching (FRAP) of GFP-rab5 in N2a cells overexpressing APPwt but does not alter endosomal rab5 activation (FRAP) in cells expressing APP at normal endogenous levels endosomes. The summary graph reflects by the percent of average fluorescence recovery at 270 s after photobleaching in each condition (*n*=20 endosomes, one endosome per cell, total 20 cells, mean±s.e.m., one-way ANOVA, Tukey's test, *P<0.05 and *P<0.01). (**d**) The PTB domain of APPL1 is required for βCTF-induced rab5 enlargement. The increased GFP-rab5 endosome size induced by APPwt and APPL1wt overexpression in N2a cells is prevented when either the APPmv mutant or the APPL1ΔPTB (PTB domain deleted APPL1) mutant is substituted for APPL1wt as the corresponding overexpressed construct. Each graph bar indicates number of measured endosomes from 20 cells (*P<0.05, mean±s.e.m., one-way ANOVA, Tukey's test). (**e**) The PTB domain of APPL1 is required for βCTF-induced GFP-rab5 activation on endosomes: FRAP in N2a cells. Overexpression of APPwt and APPL1wt in N2a cells reduces the FRAP on endosomes, indicating rab5 activation, whereas APPmv mutant or APPL1ΔPTB overexpression do not alter the FRAP relative to that in control cells. The summary graph reflects the percent of average fluorescence recovery at 270 s after photobleaching in each condition (*n*=20 endosomes, one endosome per cell, total 20 cells, mean±s.e.m., one-way ANOVA, Tukey's test, ***P<0.001).

APPL1 siRNA treatment, which significantly lowered APPL1 levels (Supplementary Figures 2c and d), reversed GTP-rab5 levels (Figure 3a), endosomal size (Figure 3b) and FRAP rate (Figure 3c) induced by APPwt overexpression, suggesting that APPL1 mediates APP-induced rab5 activation. Notably, APPL1 knockdown did not alterthe endosome size or rab5 activation in the absence of APPwt overexpression, implying that APPL1 has no major role in normal constitutive rab5 function (Figures 3b and c), which is consistent with the previous findings.^29, 39^ Furthermore, APPL1 knockdown by using siRNA did not alter Aβ formation (Supplementary Figures 3a and b). Combined overexpression of APPwt and APPL1wt in N2a cells also induced endosomal enlargement and decreased FRAP rate compared with non-transfected control N2a cells (Figures 3d and e). However, coexpression of APPmv with APPL1wt or APPwt with APPL1ΔPTB had no effect on these endosomal functions (Figures 3d and e). These data indicate that βCTF-mediated rab5 activation is dependent on APPL1. Although APPL1 shares high sequence homology with its isoform APPL2, proteins have been identified that selectively bind to APPL1 but not to APPL2.^40, 41^ This differential binding is possible because APPL1 has additional regions that are absent in APPL2.^40, 41^ Substantial reduction of APPL2 levels had no effect on endosomal enlargement in N2a cells overexpressing APPwt (Supplementary Figure 4), suggesting that βCTF-induced endosomal changes are selectively mediated by APPL1.

### APPL1 mediates βCTF-induced impairment of endosomal transport

**Figure 4:**
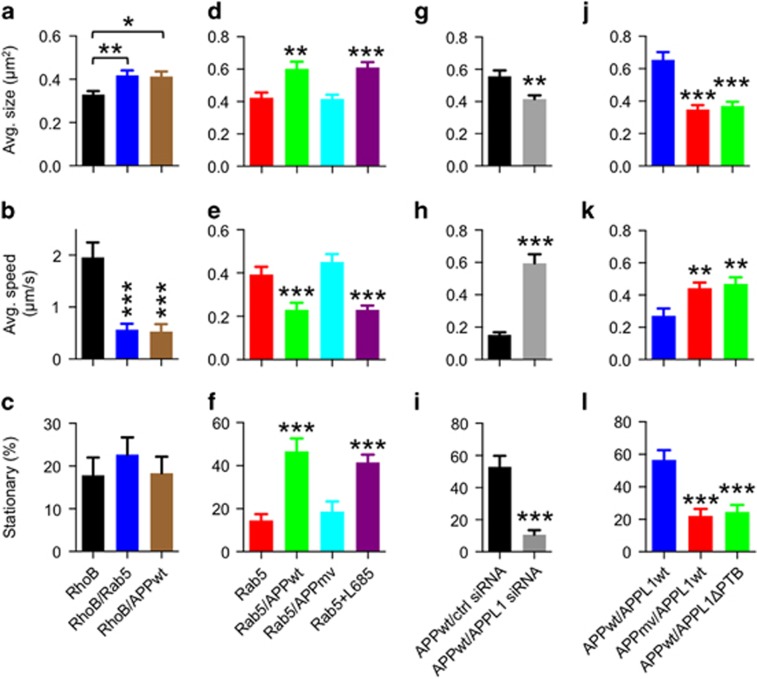
APPL1 (adaptor protein containing pleckstrin homology domain, phosphotyrosine binding domain and leucine zipper motif) mediates β-cleaved carboxy-terminal fragment of APP (βCTF)-induced enlargement and impaired transport of endosomes in neurons. (**a**) Overexpression of wild-type human APP (APPwt) or rab5 increases average cross-sectional areas of individual rhoB-positive endosomes in primary cultures of mouse cortical neurons (one-way analysis of variance (ANOVA), Tukey's test, *P<0.05 and **P<0.01, respectively). (**b**) Rab5 or APPwt overexpression reduces average transport velocities of rhoB-positive endosomes in cultured neurons (one-way ANOVA, Tukey's test, ***P<0.001). (**c**) Rab5 or APPwt overexpression has no effect on transport interruption of rhoB-positive endosomes in cultured neurons. (**d–f**) Raising βCTF levels by APPwt overexpression or exposure to the γ-secretase inhibitor L685 458 (L685) in cultured neurons increases average cross-sectional area of rab5-positive endosomes (**d**), decreases average speeds of rab5-positive endosomes (**e**) and increases numbers of stationary rab5 endosomes (**f**) (one-way ANOVA, Tukey's test, **P<0.01 and ***P<0.001). Overexpressing the β-secretase cleavage-incompetent APPmv mutant does not alter these parameters (**d–f**). (**g–i)** Treatment with APPL1 small interfering RNA (siRNA) but not a control scrambled siRNA prevents from the increased cross-sectional area of rab5 endosomes (**g**), the reduced rab5 endosome transport velocity (**h**) and the transport interruption of rab5 endosomes (i.e. increased stationary endosome number) (**i**) that are induced in primary cultures of mouse cortical neurons by APPwt overexpression (unpaired two-tailed *t*-test, **P<0.01 and ***P<0.001). (**j–l**) The PTB domain of APPL1 is required for βCTF-induced endosome alterations in primary cortical neurons. Increased endosomal cross-sectional area (**j**), lowered transport rate of endosomes (**k**) and transport interruption of rab5 endosomes (elevated stationary endosomes) (**l**) are induced by overexpressing APPwt and APPL1wt (one-way ANOVA, Tukey's test, **P<0.01 and ***P<0.001) but are not induced by overexpression of either APPL1wt with APPmv or APPwt with APPL1ΔPTB (PTB domain deleted APPL1 mutant). Results are presented as mean±s.e.m.

Because neurotrophic endosome signaling is dependent on microtubule-based retrograde axonal transport and is impaired in some neurodegenerative diseases, including mouse models of DS,^11^ we analyzed how axonal transport of endosomes is affected by overexpression of APPwt and/or rab5 in cultured mouse cortical neurons. To monitor endosome behavior directly, we transfected neurons with GFP-rhoB, a small GTPase located on both early and late endosomes. Expression of rab5 or APPwt significantly increased the average size of GFP-rhoB endosomes in an AD-like pattern^27^ (Figure 4a and Supplementary Table 1). Dynamic behaviors of GFP-rab5 endosomes captured from time-lapse images and displayed on kymographs (Supplementary Figure 5) revealed a pattern of predominantly short-range bidirectional movements contrasting with the long-range retrograde movements of rab7-positive late endosomes.^42^ Unlike rab5 endosomes, GFP-rhoB endosomes remained fluorescent after acquiring rab7 and displayed both transport patterns as expected (Supplementary Figure 5). Overexpression of either rab5 or APPwt markedly slowed the average velocity of GFP-rhoB endosomes (Figure 4b and Supplementary Table 1) but did not induce the extended pausing (stationary behavior during a 250 s observation) compared with neurons not overexpressing rab5 or APPwt (Figure 4c and Supplementary Table 1). By comparison, combined overexpression of APPwt and GFP-rab5 induced much greater endosome enlargement and endosome slowing and also increased proportions of endosomes that remained paused for longer than 250 s (stationary behavior) (Figures 4d and f). These effects approached the magnitude induced by expressing the constitutively active Q79L rab5 mutant (Supplementary Table 1). Further supporting the specificity of these APP effects, overexpression of transferrin receptor, a single-pass membrane protein that undergoes endocytosis, exerted no effects on endosome size or transport (Supplementary Table 1). Notably, overexpression of the β-cleavage-incompetent APPmv mutant in neurons altered neither endosome size nor transport, whereas elevating βCTF by inhibiting γ-secretase with L685 458 induced endosomal enlargement and disrupted endosome transport (Figures 4d and f and Supplementary Table 1), implicating βCTF as the critical factor driving these APP-related pathological effects. We can also exclude general transport failure as a basis for endosome transport slowing after APPwt overexpression because mitochondrial transport, monitored by DsRed-Mito, was unaltered (Supplementary Table 2).

APPL1 depletion using APPL1 siRNA reversed endosome enlargement and completely rescued endosome transport defects in neurons transfected with APPwt and GFP-rab5, whereas a control scrambled siRNA had no effect (Figures 4g and i and Supplementary Table 1), indicating that APPL1-mediated APP-induced endosomal trafficking defects. Co-transfection of APPwt and APPL1wt significantly increased endosomal sizes and impaired transport, but APPwt/APPL1ΔPTB did not (Figures 4j and l and Supplementary Table 1), suggesting that the PTB domain of APPL1 is required for APP-induced impairment of endosomal transport. Notably, we found that, under experimental conditions, stationary endosomes were consistently larger (>0.5 μm^2^) compared with moving endosomes, which were on average smaller (<0.5 μm^2^) (Supplementary Figure 6), consistent with considerable evidence that axonal transport rate is negatively influenced by increasing vesicle volume beyond a normal limit because of steric hindrance and cargo drag.^43, 44, 45^ Taken together, these data show that βCTF/APPL1-mediated rab5 activation impairs endosomal transport and can be considered a significant pathogenic factor in multiple neurodegenerative diseases, including AD and DS.^11, 46^

### APPL1 siRNA rescues endosomal pathobiology in DS fibroblasts

**Figure 5:**
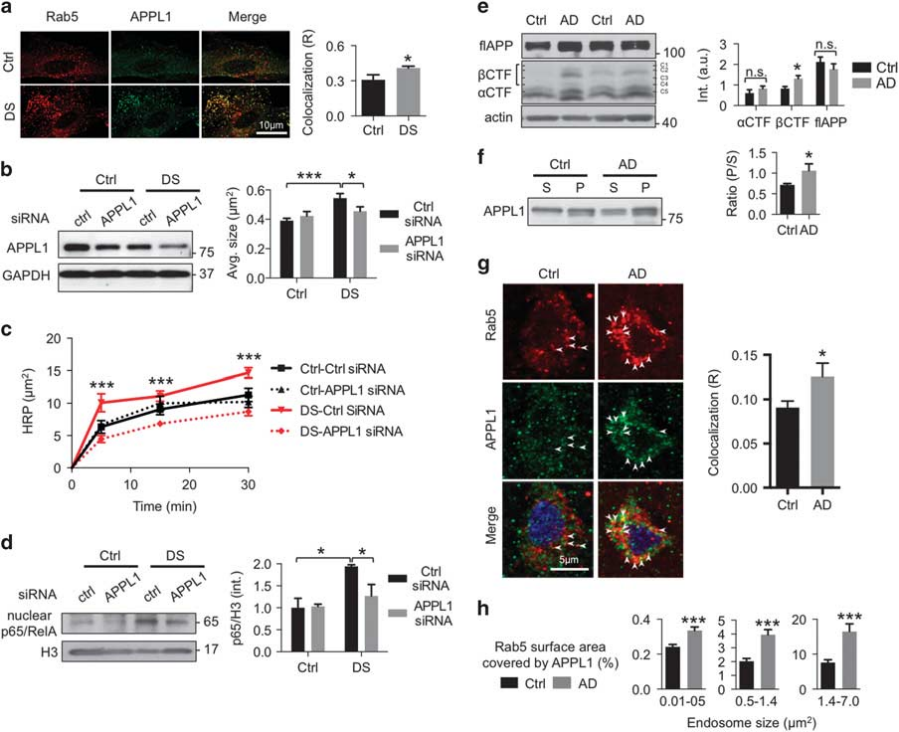
APPL1 (adaptor protein containing pleckstrin homology domain, phosphotyrosine binding domain and leucine zipper motif)- and βCTF (β-cleaved carboxy-terminal fragment of APP)-dependent endosomal abnormalities in Down syndrome (DS) fibroblasts and Alzheimer's disease (AD) brain. (**a**) Greater APPL1 colocalization with rab5 in DS fibroblasts compared with control (ctrl) fibroblasts is seen by immunocytochemistry as shown in representative images of cells double immunolabeled with antibodies to APPL1 (green) and rab5 (red). The graph shows a higher APPL1 and rab5 colocalization coefficient (R) in DS fibroblasts, as calculated by Pearson's correlation coefficient in 30 DS and 30 control cells (mean±s.e.m., unpaired two-tailed *t*-test *P<0.05). (**b**) APPL1 mediates endosomal enlargement in DS cells. Treatment with APPL1 small interfering RNA (siRNA), but not scrambled control siRNA, blocks the increase in cross-sectional area of endosomes in DS fibroblasts but has no effect on normal endosomes in age-matched 2*N* ctrl fibroblasts (*n*=30 cells, one-way analysis of variance (ANOVA), Tukey's test, mean±s.e.m., *P<0.05 and ***P<0.001). A representative blot with a glyceraldehyde 3-phosphate dehydrogenase (GAPDH) loading control and graphic quantitation from three separate experiments are shown. (**c**) APPL1 mediates the abnormally elevated endocytosis in DS fibroblasts.^7^ Fluorescent horseradish peroxidase (HRP)-positive puncta are higher in DS fibroblasts at 30 min after the addition of HRP to the medium compared with control cells and are reduced in DS cells treated with APPL1 siRNA compared with cells treated with scrambled (ctrl) siRNA, whereas APPL1 siRNA has no effect on HRP uptake in control fibroblasts. Quantitative analysis of fluorescence at 0, 5, 15 and 30 min after the addition of HRP to the medium shows significantly reduced HRP uptake after APPL1 siRNA compared with cells treated with scrambled (ctrl) siRNA (ctrl–ctrl-siRNA; 15, 15, 20 and 19 cells; ctrl-siRNA: 16, 28, 17 and 25 cells; DS-ctrl-siRNA: *n*=26, 10, 18 and 36 cells; DS-APPL1 siRNA: *n*=12, 16, 39 and 42 cells in 0, 5, 15 and 30 min from two experiments, mean±s.e.m., one-way ANOVA, Tukey's test, mean±s.e.m., ***P<0.001). (**d**) APPL1 mediates abnormally increased nuclear factor-κB (NF-κB) pathway activation in DS fibroblasts. Functional evidence for APPL1-mediated rab5 activation on endosomes is reflected in greater APPL1/rab5-endosomal-mediated nuclear localization of the NF-κB p65 subunit (p65/RelA).^16^ Levels of nuclear p65/RelA normalized to nuclear histone H3 as a loading control (third lane) are elevated in DS fibroblasts compared with those in control (ctrl) cells (first lane). This elevation is reversed by APPL1 siRNA (fourth lane) but not scrambled (ctrl) siRNA as shown on an immunoblot representative of three experiments quantified in the graph (mean±s.e.m., two-way ANOVA with interaction, *P<0.05). (**e**) βCTF levels, but not full-length APP (flAPP) or αCTF levels, are significantly elevated in AD brain compared with age-matched control (ctrl) brain, as shown on an immunoblot representative of three experiments quantified in the graph. C1/6.1 antibody was used to measure βCTF and αCTF, which are normalized to the level of APP (*n*=13 control and 13 AD brains, mean±s.e.m., unpaired two-tailed *t*-test, *P<0.05). Identities of βCTF (C1–C4) and αCTF (C5) were confirmed using 6E10 (data not shown). Actin is used as a loading control. (**f**) Membrane-associated APPL1 levels are increased in AD brains compared with control (ctrl) brains as shown on an immunoblot representative of three experiments quantified in the graph as a ratio of membrane-associated APPL1 in a 50 μg pellet (P) of brain homogenate to the APPL1 level in the 50 μg supernatant (S) (*n*=13 control and 13 AD brains, mean±s.e.m., unpaired two-tailed *t*-test, *P<0.05). (**g**) APPL1 association with rab5-positive endosomes is increased in AD brain. Double immunofluorescence labeling confirms rab5-positive endosome enlargement in neocortical pyramidal neurons of AD brain^8^ and demonstrates greater APPL1 colocalization (arrowheads) in these enlarged endosomes as compared with neuropathologically normal control brains. The graph shows a higher APPL1 and rab5 colocalization coefficient (R) in AD brains, as calculated by Pearson's correlation coefficient (*n*=90 cells, mean±s.e.m., unpaired two-tailed *t*-test, *P<0.05). (**h**) Recruitment of APPL1 to rab5 endosomes is higher in neuronal endosomes of AD brain compared with that in control (ctrl) brain, as reflected by an increased percent of rab5 endosome surface area occupied by APPL1-immunoreactive signal quantified from images similar to those in panel **g**. Comparison of rab5 endosomes of different size ranges demonstrates increased APPL1 colocalization on endosomes of greater size in AD brains (*n*=90 cells; 3824, 408 and 224 endosomes for control, 3194, 269 and 156 endosomes for AD in each size bin, mean±s.e.m., unpaired two-tailed *t*-test, ***P<0.001).

We next investigated the pathogenic importance of APPL1 alterations in fibroblasts from individuals with DS, where elevated βCTF induces rab5 activation and diverse endosome anomalies.^3, 8, 9^ APPL1 colocalized with rab5-positive endosomes to a significantly greater degree in DS fibroblasts compared with that in control cells (Figure 5a), consistent with APPwt overexpression in N2a cells (Supplementary Figure 2a) and suggesting that APP overexpression in DS cells recruits more APPL1 to rab5 endosomes. In addition, APPL1 siRNA knockdown in DS fibroblasts reduced APPL1 levels by about 50% (Figure 5b and Supplementary Figure 7) and restored normal endosomal size (Figure 5b) and reversed the abnormally high endocytic HRP uptake compared with control fibroblasts (Figure 5c and Supplementary Figure 8). We also found that APPL1 siRNA had no effect on endosomal size and HRP uptake in control cells (Figures 5b and c and Supplementary Figure 7). By contrast, siRNA knockdown of APPL2 did not reverse endosomal enlargement in DS cells (Supplementary Figure 9). This suggests that, as we observed in N2a cells (Supplementary Figure 4), βCTF-induced rab5 activation in DS fibroblasts is mediated specifically by APPL1.

To establish an impact of APPL1-mediated rab5 activation on downstream endosomal signaling, we investigated a key aspect of NF-κB signaling, the nuclear translocation of the transcriptional activator p65/RelA, which was recently shown to require rab5-dependent endosomal recruitment of APPL1.^16^ NF-κB pathway activation is reported to occur in various neurological diseases, including AD and DS.^21, 47^ Immunoblot analysis revealed that p65/RelA levels were elevated 75% in the nuclear fraction of DS fibroblasts (Figure 5d), as seen previously.^21^ Knockdown of APPL1 using siRNA, however, significantly reversed this elevation. Collectively, these data establish that APPL1 is essential for APP/βCTF-induced rab5 activation and pathological endosome-related NF-κB signaling in DS fibroblasts.

### βCTF and endosomal APPL1 levels are elevated in AD brains

We further investigated the relevance of APPL1 to neuronal endosome anomalies in late-onset AD.^8^ APP levels have been found to be normal in human AD brain,^48^ but β-secretase activity is reported to be increased,^49^ suggesting a higher rate of βCTF generation, although comparative βCTF levels in AD and matched controls have not been reported. We observed that βCTF levels, assayed as a ratio with flAPP, were significantly elevated in the AD cerebral cortex (Figure 5e), whereas levels of flAPP and αCTF were not significantly altered (Figure 5e), and similar results were also observed in brains of both Ts2 DS model mice and older adult DS individuals (unpublished data). In addition, the membrane association of APPL1 was significantly increased in AD brains (Figure 5f) and in older adult DS brains (unpublished data). No influences of post-mortem variables and demographics (e.g. age and gender) were detected (Supplementary Table 3). Furthermore, a quantitative double-immunofluorescence labeling analysis of AD and control neocortex with rab5 and APPL1 antibodies revealed a significantly higher colocalization of APPL1 with rab5 endosomes in neurons of cortical layers III and V (Figure 5g). Notably, APPL1 colocalization with rab5 endosomes was increased with greater endosome size in AD brains. APPL1 recruitment to rab5 endosomes was significantly higher in AD brains and was most abnormal (>2-fold compared with control) on the abnormally large rab5 endosomes (>0.5 μm^2^) (Figure 5h). Thus, as in DS, elevated βCTF levels in AD brain are linked to the abnormal endosomal recruitment of APPL1, rab5 upregulation and characteristic endosome anomalies of early AD.

## Discussion

Our studies define a novel βCTF-dependent pathogenic pathway that accounts for the signature endosome pathology in AD appearing at early stages of the disease and accord with growing evidence implicating the genes regulating endocytosis as frequent negative risk factors for AD.^4, 5, 6, 7^ βCTF has neurotoxic properties not dependent on cleavage to Aβ.^3, 4, 50, 51^ βCTF overexpression in transgenic mice and CTF elevations in a mouse model of Danish dementia^52^ induce age-dependent neurodegeneration and cognitive impairment.^53, 54^ BACE1 overexpression in mice, which raises brain βCTF levels and lowers Aβ, has similar effects.^55^ Importantly, our finding that βCTF levels are elevated in sporadic AD brain reveals how a βCTF-driven mechanism may apply to late-onset AD. In addition, rab5 endosome dysfunction can be potentially promoted further by multiple AD-related factors, including increased BACE1 expression and activity,^49, 56, 57^ inheritance of the ApoE E4 allele,^27^ lowered Vps35^58^ and altered handling of cholesterol, which increase BACE1 activity and βCTF levels.^59, 60, 61, 62^

Endosome anomalies are among the earliest disease-specific neuronal responses in AD and DS.^63^ In addition to βCTF of APP, other genetic factors have been reported to induce endosomal dysfunction in AD and DS, including synaptojanin1^64^ and Vps34, a class III phosphoinositide 3-kinase,^65^ and possibly their relationships with rab5-dependent or -independent mechanisms deserve further investigation. Rab5-mediated acceleration of endocytosis and endosome fusion cause endosomes to enlarge and selectively disrupt their transport in neurons, possibly mediated by steric hindrance^43, 44, 45^ or rab5-mediated activation of Vps34.^19^ Substantial transport slowing of enlarged vesicles is seen under various experimental conditions^44^ and the tipping point for axonal transport slowing of organelles occurs when vesicular expansion reaches sizes above 0.5 μm^2^ (unpublished data). In addition, Vps34 activity, which is modulated by rab5, has been implicated in endosomal transport and endosome-related disease pathogenesis.^19, 65^ Although the exact mechanism of trafficking failure of enlarged endosomes needs further investigation, rab5 overactivation promoted by a βCTF–APPL1 interaction provides a plausible basis for the APP-dependent failure of retrograde neurotrophin signaling by endosomes previously implicated in the neurodegeneration of cholinergic neurons in DS mouse model.^11^ Disrupted transport of endosomes and their focal accumulation in axons mainly with autophagic vacuoles, which likely reflects attempted endosome clearance by autophagy, leads to neuritic dystrophy resembling that observed at early stages of AD.^46, 63^ Furthermore, accelerated endocytosis causes endocytic cargos to accumulate within enlarged late endosomes^9^ and impairs lysosome functions in a βCTF-dependent manner (unpublished data). Therefore, the pathological consequences of βCTF-induced pathological rab5 activation may be diverse and include, as shown here, selective impairment of endosome transport in neurons—a possible basis for impaired endosomal signaling linked to neurodegeneration,^9, 46^ and alteration of NF-κB pathway signaling via endosomes—an effect known to promote neuronal apoptosis in some contexts.^47^

We present multiple lines of evidence from neuronal APP models and DS patient fibroblasts and additional support from analyses of Alzheimer brains, indicating that APPL1 is the mediator of βCTF-induced rab5 activation, which underlies the very early-appearing endocytic dysfunction in AD. This mechanism is consistent with the reported ability of APPL1 to increase rab5 localization on enlarged endosomes.^29^ Our data suggest that APPL1 recruited by βCTF could stabilize rab5 in its active GTP state and slow its loss from endosomes. We have previously shown that the levels of αCTF do not influence rab5 upregulation in DS fibroblasts,^3^ and, consistent with this finding, we observed that APPL1 antibodies did not pull down appreciable αCTF even though it contains the same APPL1-interacting domain as βCTF. In this regard, αCTF is mainly generated at the cell surface^66^ and APPL1 is excluded from the plasma membrane.^13, 15^ By contrast, βCTF is more abundantly generated on endosomes^66^ where a distinctive set of signaling ligands may contribute to its selective interaction with APPL1. In this regard, APP intracellular domain generated from βCTF by γ-secretase on endosomes can be stabilized by binding to other proteins, such as JIP-1, and translocated to the nucleus,^67^ whereas APP intracellular domain produced from cell surface αCTF is degraded into smaller fragments.^67^ To further support these earlier data, we showed that inhibition of endocytosis decreases APPL1–βCTF interaction and increases the levels of αCTF, but not its interaction with APPL1 (Figure 2f), consistent with its relative inaccessibity to endosomal proteins, such as APPL1. Finally, we show here that αCTFs levels do not influence endosomal pathology in cells (Figure 1c), as previously seen in DS fibroblasts.^3^ Thus, we suggest that selective APPL1 with βCTF may partly reflect to the differential compartmentalization of full-length, α-cleaved and β-cleaved APP, although it is possible that unidentified proteins interacting with βCTF and APPL1 on endosomes may enhance specificity of this interaction.

Notably, APPL1 on endosomes is normally replaced by phosphatidylinositol 3-triphosphate-binding proteins, such as EEA1 (early endosome antigen 1).^15^ The reduced phosphatidylinositol 3-triphosphate levels seen in AD brain^65^ could contribute to APPL1 elevation on endosomes. APPL1 overexpression mimicked the pathological effects of βCTF on endosome morphology and function,^29^ whereas APPL1 knockdown prevented these effects and reversed them in DS cells. Furthermore, APPL1 positively regulates TNFα-independent NF-κB activation via APPL1/rab5 endosomes.^68^ We used this phenomenon as additional functional evidence of increased APPL1-dependent activity on endosomes in DS beyond its effect on stabilizing active rab5 on endosomes. Increased NF-κB activation in several major neurological diseases,^69, 70^ including AD,^42^ is implicated in neurodegenerative mechanisms, although its range of effects on neuronal vulnerability are complex. Importantly, we observed that APPL1 knockdown minimally affected endocytosis by cells that express endogenous APP at normal levels,^29^ implying that βCTF/APPL1-dependent rab5 activation is not essential for normal constitutive endocytosis but is instead a superimposed pathological phenomenon. Moreover, APPL1 overexpression did not affect βCTF levels (Supplementary Figure 10), suggesting that βCTF, not APPL1, is the more upstream initiator of endosomal pathology in both AD and DS. These observations raise the possibility that the pathogenic endocytosis mediated by elevated βCTF–APPL1 could be modulated therapeutically at multiple possible targets without altering vital functions of basal endocytosis.

## Supplementary Material

Supplementary Informations

## References

[bib1] Pimplikar SW, Nixon RA, Robakis NK, Shen J, Tsai LH. Amyloid-independent mechanisms in Alzheimer's disease pathogenesis. J Neurosci 2010; 30: 14946–14954. 21068297 10.1523/JNEUROSCI.4305-10.2010 PMC3426835

[bib2] Krstic D, Knuesel I. Deciphering the mechanism underlying late-onset Alzheimer disease. Nat Rev Neurol 2013; 9: 25–34. 23183882 10.1038/nrneurol.2012.236

[bib3] Jiang Y, Mullaney KA, Peterhoff CM, Che S, Schmidt SD, Boyer-Boiteau A et al. Alzheimer's-related endosome dysfunction in Down syndrome is A{beta}-independent but requires APP and is reversed by BACE-1 inhibition. Proc Natl Acad Sci USA 2010; 107: 1630–1635. 20080541 10.1073/pnas.0908953107 PMC2824382

[bib4] Israel MA, Yuan SH, Bardy C, Reyna SM, Mu Y, Herrera C et al. Probing sporadic and familial Alzheimer's disease using induced pluripotent stem cells. Nature 2012; 482: 216–220. 22278060 10.1038/nature10821 PMC3338985

[bib5] Rhinn H, Fujita R, Qiang L, Cheng R, Lee JH, Abeliovich A. Integrative genomics identifies APOE epsilon4 effectors in Alzheimer's disease. Nature 2013; 500: 45–50. 23883936 10.1038/nature12415

[bib6] Nixon RA. The role of autophagy in neurodegenerative disease. Nat Med 2013; 19: 983–997. 23921753 10.1038/nm.3232

[bib7] Ginsberg SD, Alldred MJ, Counts SE, Cataldo AM, Neve RL, Jiang Y et al. Microarray analysis of hippocampal CA1 neurons implicates early endosomal dysfunction during Alzheimer's disease progression. Biol Psychiatry 2010; 68: 885–893. 20655510 10.1016/j.biopsych.2010.05.030 PMC2965820

[bib8] Cataldo AM, Peterhoff CM, Troncoso JC, Gomez-Isla T, Hyman BT, Nixon RA. Endocytic pathway abnormalities precede amyloid beta deposition in sporadic Alzheimer's disease and Down syndrome: differential effects of APOE genotype and presenilin mutations. Am J Pathol 2000; 157: 277–286. 10880397 10.1016/s0002-9440(10)64538-5 PMC1850219

[bib9] Cataldo AM, Mathews PM, Boiteau AB, Hassinger LC, Peterhoff CM, Jiang Y et al. Down syndrome fibroblast model of Alzheimer-related endosome pathology: accelerated endocytosis promotes late endocytic defects. Am J Pathol 2008; 173: 370–384. 18535180 10.2353/ajpath.2008.071053 PMC2475775

[bib10] Laifenfeld D, Patzek LJ, McPhie DL, Chen Y, Levites Y, Cataldo AM et al. Rab5 mediates an amyloid precursor protein signaling pathway that leads to apoptosis. J Neurosci 2007; 27: 7141–7153. 17611268 10.1523/JNEUROSCI.4599-06.2007 PMC6794581

[bib11] Salehi A, Delcroix JD, Belichenko PV, Zhan K, Wu C, Valletta JS et al. Increased App expression in a mouse model of Down's syndrome disrupts NGF transport and causes cholinergic neuron degeneration. Neuron 2006; 51: 29–42. 16815330 10.1016/j.neuron.2006.05.022

[bib12] Ng EL, Tang BL. Rab GTPases and their roles in brain neurons and glia. Brain Res Rev 2008; 58: 236–246. 18485483 10.1016/j.brainresrev.2008.04.006

[bib13] Miaczynska M, Christoforidis S, Giner A, Shevchenko A, Uttenweiler-Joseph S, Habermann B et al. APPL proteins link Rab5 to nuclear signal transduction via an endosomal compartment. Cell 2004; 116: 445–456. 15016378 10.1016/s0092-8674(04)00117-5

[bib14] Zhu G, Chen J, Liu J, Brunzelle JS, Huang B, Wakeham N et al. Structure of the APPL1 BAR-PH domain and characterization of its interaction with Rab5. EMBO J 2007; 26: 3484–3493. 17581628 10.1038/sj.emboj.7601771 PMC1933402

[bib15] Zoncu R, Perera RM, Balkin DM, Pirruccello M, Toomre D, De Camilli P. A phosphoinositide switch controls the maturation and signaling properties of APPL endosomes. Cell 2009; 136: 1110–1121. 19303853 10.1016/j.cell.2009.01.032 PMC2705806

[bib16] Hupalowska A, Pyrzynska B, Miaczynska M. APPL1 regulates basal NF-kappaB activity by stabilizing NIK. J Cell Sci 2012; 125: 4090–4102. 22685329 10.1242/jcs.105171 PMC3482318

[bib17] Bohdanowicz M, Balkin DM, De Camilli P, Grinstein S. Recruitment of OCRL and Inpp5B to phagosomes by Rab5 and APPL1 depletes phosphoinositides and attenuates Akt signaling. Mol Biol Cell 2012; 23: 176–187. 22072788 10.1091/mbc.E11-06-0489 PMC3248896

[bib18] Lee JR, Hahn HS, Kim YH, Nguyen HH, Yang JM, Kang JS et al. Adaptor protein containing PH domain, PTB domain and leucine zipper (APPL1) regulates the protein level of EGFR by modulating its trafficking. Biochem Biophys Res Commun 2011; 415: 206–211. 22037462 10.1016/j.bbrc.2011.10.064

[bib19] Stenmark H. Rab GTPases as coordinators of vesicle traffic. Nat Rev Mol Cell Biol 2009; 10: 513–525. 19603039 10.1038/nrm2728

[bib20] Cossec JC, Simon A, Marquer C, Moldrich RX, Leterrier C, Rossier J et al. Clathrin-dependent APP endocytosis and Abeta secretion are highly sensitive to the level of plasma membrane cholesterol. Biochim Biophys Acta 2010; 1801: 846–852. 20580937 10.1016/j.bbalip.2010.05.010

[bib21] Engidawork E, Gulesserian T, Seidl R, Cairns N, Lubec G. Expression of apoptosis related proteins: RAIDD, ZIP kinase, Bim/BOD, p21, Bax, Bcl-2 and NF-kappaB in brains of patients with Down syndrome. J Neural Transm Suppl 2001; 61: 181–192. 10.1007/978-3-7091-6262-0_14 11771742

[bib22] Lee S, Sato Y, Nixon RA. Lysosomal proteolysis inhibition selectively disrupts axonal transport of degradative organelles and causes an Alzheimer's-like axonal dystrophy. J Neurosci 2011; 31: 7817–7830. 21613495 10.1523/JNEUROSCI.6412-10.2011 PMC3351137

[bib23] Vieira OV, Bucci C, Harrison RE, Trimble WS, Lanzetti L, Gruenberg J et al. Modulation of Rab5 and Rab7 recruitment to phagosomes by phosphatidylinositol 3-kinase. Mol Cell Biol 2003; 23: 2501–2514. 12640132 10.1128/MCB.23.7.2501-2514.2003 PMC150733

[bib24] Bucci C, Parton RG, Mather IH, Stunnenberg H, Simons K, Hoflack B et al. The small GTPase rab5 functions as a regulatory factor in the early endocytic pathway. Cell 1992; 70: 715–728. 1516130 10.1016/0092-8674(92)90306-w

[bib25] Stenmark H, Parton RG, Steele-Mortimer O, Lutcke A, Gruenberg J, Zerial M. Inhibition of rab5 GTPase activity stimulates membrane fusion in endocytosis. Embo J 1994; 13: 1287–1296. 8137813 10.1002/j.1460-2075.1994.tb06381.x PMC394944

[bib26] Citron M, Teplow DB, Selkoe DJ. Generation of amyloid beta protein from its precursor is sequence specific. Neuron 1995; 14: 661–670. 7695913 10.1016/0896-6273(95)90323-2

[bib27] Cataldo AM, Barnett JL, Pieroni C, Nixon RA. Increased neuronal endocytosis and protease delivery to early endosomes in sporadic Alzheimer's disease: neuropathologic evidence for a mechanism of increased beta-amyloidogenesis. J Neurosci 1997; 17: 6142–6151. 9236226 10.1523/JNEUROSCI.17-16-06142.1997 PMC6568334

[bib28] Tamayev R, Zhou D, D'Adamio L. The interactome of the amyloid beta precursor protein family members is shaped by phosphorylation of their intracellular domains. Mol Neurodegener 2009; 4: 28. 19602287 10.1186/1750-1326-4-28 PMC2723102

[bib29] Chial HJ, Wu R, Ustach CV, McPhail LC, Mobley WC, Chen YQ. Membrane targeting by APPL1 and APPL2: dynamic scaffolds that oligomerize and bind phosphoinositides. Traffic 2008; 9: 215–229. 18034774 10.1111/j.1600-0854.2007.00680.x PMC3810297

[bib30] Ogawa A, Yamazaki Y, Nakamori M, Takahashi T, Kurashige T, Hiji M et al. Characterization and distribution of adaptor protein containing a PH domain, PTB domain and leucine zipper motif (APPL1) in Alzheimer's disease hippocampus: an immunohistochemical study. Brain Res 2012; 1494: 118–124. 23246927 10.1016/j.brainres.2012.12.010

[bib31] Perez RG, Soriano S, Hayes JD, Ostaszewski B, Xia W, Selkoe DJ et al. Mutagenesis identifies new signals for beta-amyloid precursor protein endocytosis, turnover, and the generation of secreted fragments, including Abeta42. J Biol Chem 1999; 274: 18851–18856. 10383380 10.1074/jbc.274.27.18851

[bib32] Young-Pearse TL, Bai J, Chang R, Zheng JB, LoTurco JJ, Selkoe DJ. A critical function for beta-amyloid precursor protein in neuronal migration revealed by in utero RNA interference. J Neurosci 2007; 27: 14459–14469. 18160654 10.1523/JNEUROSCI.4701-07.2007 PMC6673432

[bib33] Borg JP, Ooi J, Levy E, Margolis B. The phosphotyrosine interaction domains of X11 and FE65 bind to distinct sites on the YENPTY motif of amyloid precursor protein. Mol Cell Biol 1996; 16: 6229–6241. 8887653 10.1128/mcb.16.11.6229 PMC231626

[bib34] Chen Y, Liu W, McPhie DL, Hassinger L, Neve RL. APP-BP1 mediates APP-induced apoptosis and DNA synthesis and is increased in Alzheimer's disease brain. J Cell Biol 2003; 163: 27–33. 14557245 10.1083/jcb.200304003 PMC2173435

[bib35] Beel AJ, Sakakura M, Barrett PJ, Sanders CR. Direct binding of cholesterol to the amyloid precursor protein: an important interaction in lipid-Alzheimer's disease relationships? Biochim Biophys Acta 2010; 1801: 975–982. 20304095 10.1016/j.bbalip.2010.03.008 PMC2886191

[bib36] Zhu L, Su M, Lucast L, Liu L, Netzer WJ, Gandy SE et al. Dynamin 1 regulates amyloid generation through modulation of BACE-1. PLoS One 2012; 7: e45033. 23024787 10.1371/journal.pone.0045033 PMC3443198

[bib37] Sisodia SS. Beta-amyloid precursor protein cleavage by a membrane-bound protease. Proc Natl Acad Sci USA 1992; 89: 6075–6079. 1631093 10.1073/pnas.89.13.6075 PMC49440

[bib38] Minopoli G, de Candia P, Bonetti A, Faraonio R, Zambrano N, Russo T. The beta-amyloid precursor protein functions as a cytosolic anchoring site that prevents Fe65 nuclear translocation. J Biol Chem 2001; 276: 6545–6550. 11085987 10.1074/jbc.M007340200

[bib39] Tan Y, You H, Wu C, Altomare DA, Testa JR. Appl1 is dispensable for mouse development, and loss of Appl1 has growth factor-selective effects on Akt signaling in murine embryonic fibroblasts. J Biol Chem 2010; 285: 6377–6389. 20040596 10.1074/jbc.M109.068452 PMC2825433

[bib40] Ryu J, Galan AK, Xin X, Dong F, Abdul-Ghani MA, Zhou L et al. APPL1 potentiates insulin sensitivity by facilitating the binding of IRS1/2 to the insulin receptor. Cell Rep 2014; 7: 1227–1238. 24813896 10.1016/j.celrep.2014.04.006 PMC4380268

[bib41] Erdmann KS, Mao Y, McCrea HJ, Zoncu R, Lee S, Paradise S et al. A role of the Lowe syndrome protein OCRL in early steps of the endocytic pathway. Dev Cell 2007; 13: 377–390. 17765681 10.1016/j.devcel.2007.08.004 PMC2025683

[bib42] Deinhardt K, Salinas S, Verastegui C, Watson R, Worth D, Hanrahan S et al. Rab5 and Rab7 control endocytic sorting along the axonal retrograde transport pathway. Neuron 2006; 52: 293–305. 17046692 10.1016/j.neuron.2006.08.018

[bib43] Allen RD, Metuzals J, Tasaki I, Brady ST, Gilbert SP. Fast axonal transport in squid giant axon. Science 1982; 218: 1127–1129. 6183744 10.1126/science.6183744

[bib44] Kaasik A, Safiulina D, Choubey V, Kuum M, Zharkovsky A, Veksler V. Mitochondrial swelling impairs the transport of organelles in cerebellar granule neurons. J Biol Chem 2007; 282: 32821–32826. 17785462 10.1074/jbc.M702295200

[bib45] Yi JY, Ori-McKenney KM, McKenney RJ, Vershinin M, Gross SP, Vallee RB. High-resolution imaging reveals indirect coordination of opposite motors and a role for LIS1 in high-load axonal transport. J Cell Biol 2011; 195: 193–201. 22006948 10.1083/jcb.201104076 PMC3198168

[bib46] Stokin GB, Lillo C, Falzone TL, Brusch RG, Rockenstein E, Mount SL et al. Axonopathy and transport deficits early in the pathogenesis of Alzheimer's disease. Science 2005; 307: 1282–1288. 15731448 10.1126/science.1105681

[bib47] Tilstra JS, Clauson CL, Niedernhofer LJ, Robbins PD. NF-kappaB in Aging and Disease. Aging Dis 2011; 2: 449–465. 22396894 PMC3295063

[bib48] Olsson A, Hoglund K, Sjogren M, Andreasen N, Minthon L, Lannfelt L et al. Measurement of alpha- and beta-secretase cleaved amyloid precursor protein in cerebrospinal fluid from Alzheimer patients. Exp Neurol 2003; 183: 74–80. 12957490 10.1016/s0014-4886(03)00027-x

[bib49] Fukumoto H, Cheung BS, Hyman BT, Irizarry MC. Beta-secretase protein and activity are increased in the neocortex in Alzheimer disease. Arch Neurol 2002; 59: 1381–1389. 12223024 10.1001/archneur.59.9.1381

[bib50] Devi L, Ohno M. Mitochondrial dysfunction and accumulation of the beta-secretase-cleaved C-terminal fragment of APP in Alzheimer's disease transgenic mice. Neurobiol Dis 2012; 45: 417–424. 21933711 10.1016/j.nbd.2011.09.001 PMC3225635

[bib51] McPhie DL, Golde T, Eckman CB, Yager D, Brant JB, Neve RL. Beta-secretase cleavage of the amyloid precursor protein mediates neuronal apoptosis caused by familial Alzheimer's disease mutations. Brain Res Mol Brain Res 2001; 97: 103–113. 11744168 10.1016/s0169-328x(01)00294-7

[bib52] Tamayev R, Matsuda S, Arancio O, D'Adamio L. Beta- but not gamma-secretase proteolysis of APP causes synaptic and memory deficits in a mouse model of dementia. EMBO Mol Med 2012; 4: 171–179. 22170863 10.1002/emmm.201100195 PMC3376850

[bib53] Oster-Granite ML, McPhie DL, Greenan J, Neve RL. Age-dependent neuronal and synaptic degeneration in mice transgenic for the C terminus of the amyloid precursor protein. J Neurosci 1996; 16: 6732–6741. 8824314 10.1523/JNEUROSCI.16-21-06732.1996 PMC6579256

[bib54] Berger-Sweeney J, McPhie DL, Arters JA, Greenan J, Oster-Granite ML, Neve RL. Impairments in learning and memory accompanied by neurodegeneration in mice transgenic for the carboxyl-terminus of the amyloid precursor protein. Brain Res Mol Brain Res 1999; 66: 150–162. 10095087 10.1016/s0169-328x(99)00014-5

[bib55] Rockenstein E, Mante M, Alford M, Adame A, Crews L, Hashimoto M et al. High beta-secretase activity elicits neurodegeneration in transgenic mice despite reductions in amyloid-beta levels: implications for the treatment of Alzheimer disease. J Biol Chem 2005; 280: 32957–32967. 16027115 10.1074/jbc.M507016200

[bib56] Stockley JH, O'Neill C. The proteins BACE1 and BACE2 and beta-secretase activity in normal and Alzheimer's disease brain. Biochem Soc Trans 2007; 35: 574–576. 17511655 10.1042/BST0350574

[bib57] Coulson DT, Beyer N, Quinn JG, Brockbank S, Hellemans J, Irvine GB et al. BACE1 mRNA expression in Alzheimer's disease postmortem brain tissue. J Alzheimer's Dis 2010; 22: 1111–1122. 20930286 10.3233/JAD-2010-101254

[bib58] Small SA, Kent K, Pierce A, Leung C, Kang MS, Okada H et al. Model-guided microarray implicates the retromer complex in Alzheimer's disease. Ann Neurol 2005; 58: 909–919. 16315276 10.1002/ana.20667

[bib59] Refolo LM, Malester B, LaFrancois J, Bryant-Thomas T, Wang R, Tint GS et al. Hypercholesterolemia accelerates the Alzheimer's amyloid pathology in a transgenic mouse model. Neurobiol Dis 2000; 7: 321–331. 10964604 10.1006/nbdi.2000.0304

[bib60] Mastrocola R, Guglielmotto M, Medana C, Catalano MG, Cutrupi S, Borghi R et al. Dysregulation of SREBP2 induces BACE1 expression. Neurobiol Dis 2011; 44: 116–124. 21726644 10.1016/j.nbd.2011.06.010

[bib61] Marquer C, Devauges V, Cossec JC, Liot G, Lecart S, Saudou F et al. Local cholesterol increase triggers amyloid precursor protein-Bace1 clustering in lipid rafts and rapid endocytosis. FASEB J 2011; 25: 1295–1305. 21257714 10.1096/fj.10-168633

[bib62] Wen L, Tang FL, Hong Y, Luo SW, Wang CL, He W et al. VPS35 haploinsufficiency increases Alzheimer's disease neuropathology. J Cell Biol 2011; 195: 765–779. 22105352 10.1083/jcb.201105109 PMC3257571

[bib63] Nixon RA. Autophagy, amyloidogenesis and Alzheimer disease. J Cell Sci 2007; 120: 4081–4091. 18032783 10.1242/jcs.019265

[bib64] Cossec JC, Lavaur J, Berman DE, Rivals I, Hoischen A, Stora S et al. Trisomy for synaptojanin1 in Down syndrome is functionally linked to the enlargement of early endosomes. Hum Mol Genet 2012; 21: 3156–3172. 22511594 10.1093/hmg/dds142 PMC3384382

[bib65] Morel E, Chamoun Z, Lasiecka ZM, Chan RB, Williamson RL, Vetanovetz C et al. Phosphatidylinositol-3-phosphate regulates sorting and processing of amyloid precursor protein through the endosomal system. Nat Commun 2013; 4: 2250. 23907271 10.1038/ncomms3250 PMC3905799

[bib66] Lee MS, Kao SC, Lemere CA, Xia W, Tseng HC, Zhou Y et al. APP processing is regulated by cytoplasmic phosphorylation. J Cell Biol 2003; 163: 83–95. 14557249 10.1083/jcb.200301115 PMC2173445

[bib67] Grimm MO, Mett J, Stahlmann CP, Haupenthal VJ, Zimmer VC, Hartmann T. Neprilysin and Abeta clearance: impact of the APP intracellular domain in NEP regulation and implications in Alzheimer's disease. Front Aging Neurosci 2013; 5: 98. 24391587 10.3389/fnagi.2013.00098 PMC3870290

[bib68] Granic I, Dolga AM, Nijholt IM, van Dijk G, Eisel UL. Inflammation and NF-kappaB in Alzheimer's disease and diabetes. J Alzheimers Dis 2009; 16: 809–821. 19387114 10.3233/JAD-2009-0976

[bib69] Trager U, Andre R, Lahiri N, Magnusson-Lind A, Weiss A, Grueninger S et al. HTT-lowering reverses Huntington's disease immune dysfunction caused by NFkappaB pathway dysregulation. Brain 2014; 137: 819–833. 24459107 10.1093/brain/awt355 PMC3983408

[bib70] Ghosh A, Roy A, Liu X, Kordower JH, Mufson EJ, Hartley DM et al. Selective inhibition of NF-kappaB activation prevents dopaminergic neuronal loss in a mouse model of Parkinson's disease. Proc Natl Acad Sci USA 2007; 104: 18754–18759. 18000063 10.1073/pnas.0704908104 PMC2141849

