# Supplementary material for: Evidence that the rab5 effector APPL1 mediates APP-βCTF-induced dysfunction of endosomes in Down syndrome and Alzheimer's disease
Source: Mol Psychiatry. 2015 Jul 21;21(5):707–16. doi: 10.1038/mp.2015.97 (PMC4721948; doi:10.1038/mp.2015.97)
Supplement: Supplementary Informations [file mp201597x1.pdf]

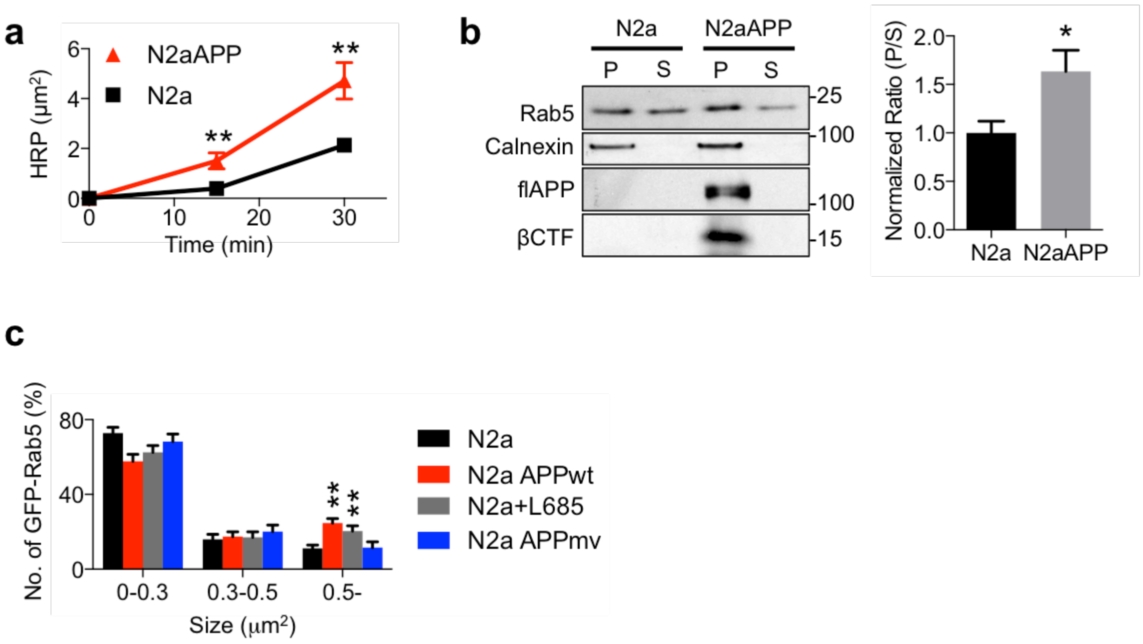

Supplementary Figure 1. βCTF-mediated rab5 activation. (a) Endocytosis is abnormally elevated by APP overexpression as shown in N2a cells stably overexpressing wild-type human APP (N2aAPP). Quantitative graphic analysis of fluorescence of internalized HRP at 15 and 30 min after HRP addition to the medium in N2aAPP cells shows significantly increased HRP uptake (n=10 cells, mean ± S.E.M., unpaired two-tailed *t*-test $^{**}p<0.01$), consistent with accelerated endocytosis shown previously in DS fibroblasts measured by transferrin, HRP, and EGF uptake[1, 2]. **(b)** APP overexpression induces rab5 activation in N2aAPP cells as evidenced by an increased level of membrane-bound rab5 (GTP-loaded active form) relative to cytosolic rab5 (GDP-bound inactive form) as measured in membrane (P) and soluble (S) fractions of N2aAPP cells versus N2a cells. Calnexin is used as a membrane marker and human flAPP and βCTF are markers for APP overexpression. The graph depicts the increased proportions of membrane-bound rab5 over soluble rab5 in the absence and presence of APPwt overexpression (n=6 experiments, mean ± S.E.M., unpaired two-tailed *t*-test, $^{*}p<0.05$). **(c)** βCTF-mediated endosomal enlargement disproportionately affects the larger endosome population. **Fig. 1c.** In quantitative distribution analyses of endosome size, overexpression of APPwt or inhibition of γ-cleavage in N2a cells causes disproportionate expansion of large endosomes ($>0.5 \mu m^2$) (n=20 cells, mean ± S.E.M., one-way ANOVA, Tukey's test, $^{**}p<0.01$) as seen previously in DS fibroblasts[1, 2].

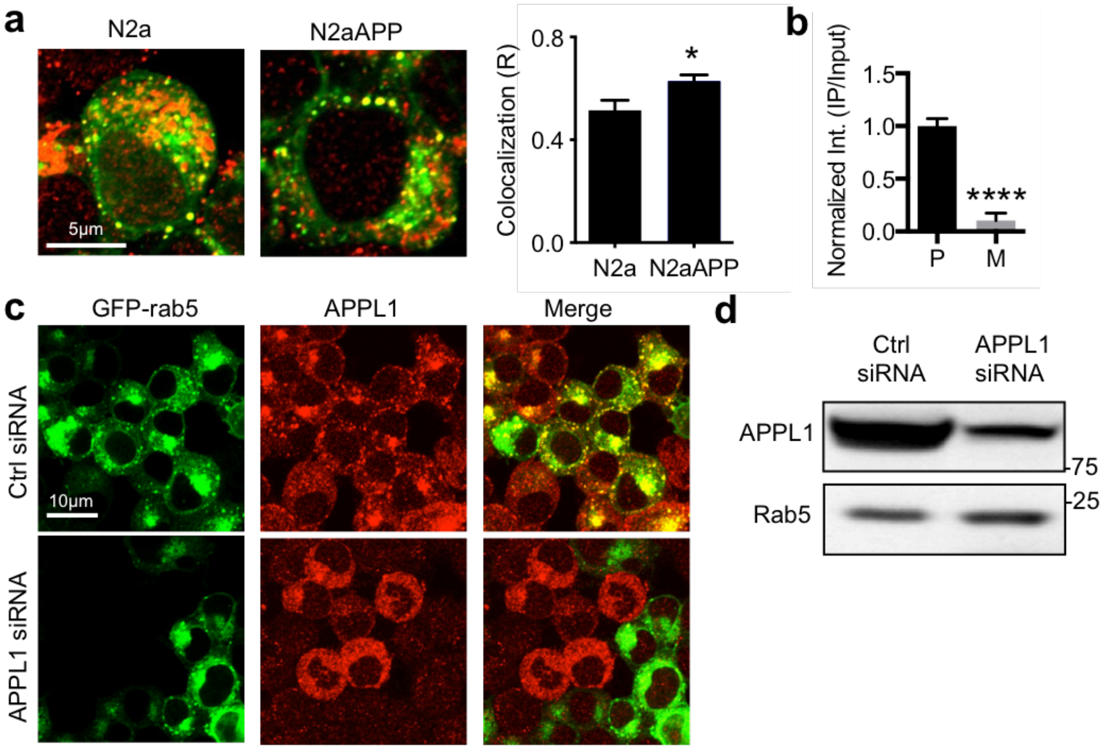

Supplementary Figure 2. APPL1 is involved in βCTF-mediated rab5 activation. (a) APP overexpression recruits more APPL1 to rab5-endosomes. APPL1 colocalization with rab5 is greater in APP overexpressed N2a cells (N2aAPP) than in control N2a cells as seen in representative images of cells double-immunolabeled with antibodies to APPL1 (red) and rab5 (green). The graph shows a higher APPL1 and rab5 colocalization (yellow) coefficient (R) in N2aAPP, as calculated by Pearson's correlation coefficient (n=20 cells, mean ± S.E.M., unpaired two-tailed *t*-test, *p<0.05). **(b)** Quantitative analysis of the interaction of APPL1 with C1/6.1 antibody in Fig. 2f shows that inhibition of endocytosis significantly decreases the interaction of APPL1 with βCTF (n=4 experiments, mean ± S.E.M., unpaired two-tailed *t*-test, ****p<0.0001). **(c-d)** APPL1 levels are significantly reduced by treating N2a cells with siRNA. Representative confocal images of APPL1 siRNA transfected cells. APPL1 siRNA or control scramble siRNA is transfected with GFP-rab5 (green) in N2a cells, and APPL1 is visualized with APPL1 antibody (red). APPL1 is significantly reduced when APPL1 siRNA is transfected with GFP-rab5 **(c)**. A representative immunoblot shows reduced APPL1 levels by siRNA in N2a cells with rab5 used as a loading control **(d)**.

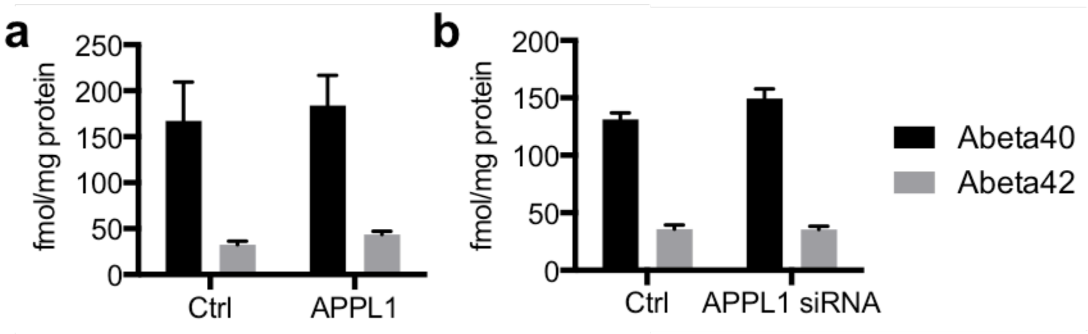

Supplementary Figure 3. APPL1 has no role on Aβ secretion. (a-b) ELISA measurements of secreted Aβ in culture media of N2aAPP transfected with APPL1 or APPL1 siRNA shows that APPL1 overexpression **(a)** or APPL1 knockdown **(b)** has no detectable effect on either Aβ40 or Aβ42 secretion (n=3 cultures duplicated).

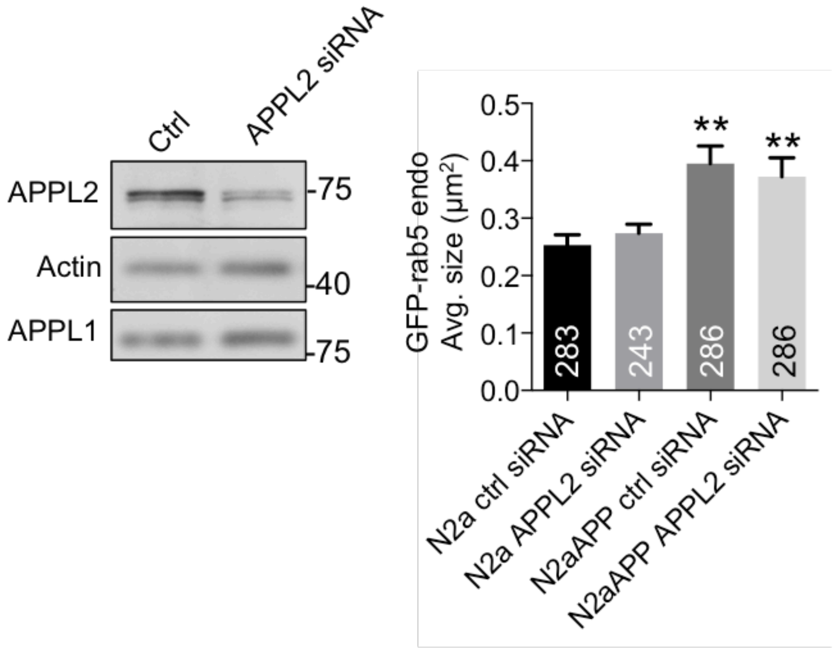

Supplementary Figure 4. APPL2 has no role in endosomal enlargement in N2aAPP cells. APPL2 siRNA significantly reduces APPL2 levels in N2a cells as shown on representative immunoblots. Actin and APPL1 are used as loading controls. Right-most panel showing quantified average size (cross-sectional area) of GFP-rab5 endosomes indicates no effect of APPL2 siRNA on APPwt-mediated endosomal enlargement in comparisons with N2a cells co-transfected with scrambled (ctrl) APPL2 siRNA in the presence or absence of APPwt overexpression (n=numbers of counted endosomes from 20 cells, mean ± S.E.M., one-way ANOVA, Tukey's test, $^{**}p<0.01$).

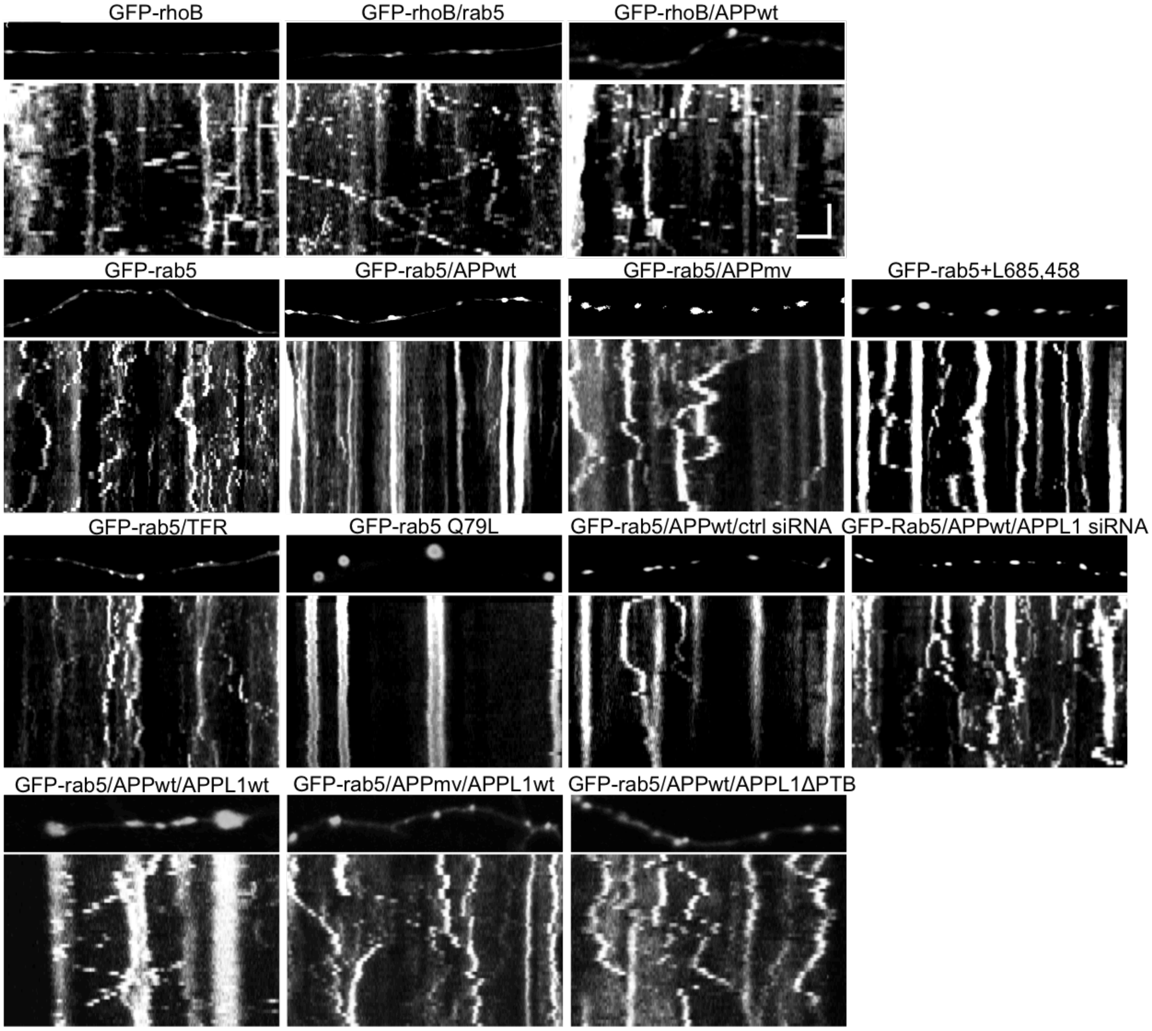

Supplementary Figure 5. Time-lapse analysis of endosome axonal transport. Representative segments of axons containing GFP-rhoB or GFP-rab5-positive endosomes (upper panels) and corresponding kymographs (bottom panels) tracking the movement of fluorescently-labeled endosomes showing dynamic behaviors of GFP-rhoB or GFP-rab5 endosomes. Rab5 endosomes show short-range bidirectional movements, while rhoB endosomes display both local and long-range retrograde movements. Scale bars in kymographs indicate 5μm of distance moved (X-axis) and 50 seconds of recording (Y-axis).

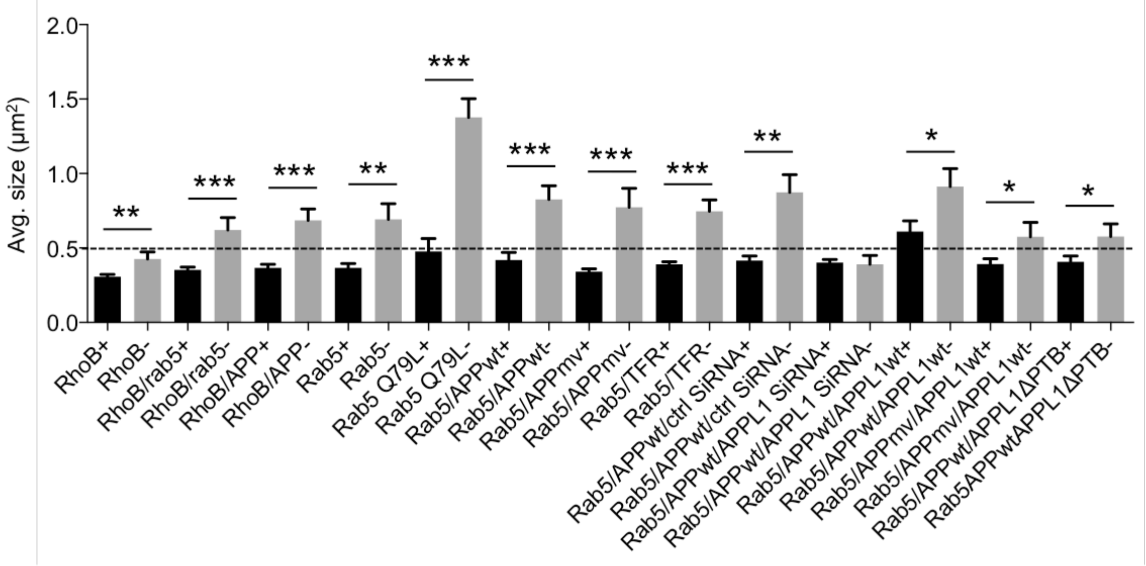

Supplementary Figure 6. Enlarged endosomes have greater interruptions of axonal transport as reflected in increased time in a stationary state. The sizes (cross-sectional areas) of GFP-rhoB and GFP-rab5–positive endosomes that display either moving (+) or entirely stationary (-) behavior in a 270 sec observation interval (as analyzed in Fig. 4**)** are shown ($<0.5\mu m^2$). (mean ± S.E.M., unpaired two-tailed *t*-test, *p<0.05, **p<0.01, and ***p<0.001). A dotted line indicates $0.5\mu m^2$. Across all conditions (with one exception), the average size of stationary endosomes is larger ($>0.5\mu m^2$) than that of motile endosomes.

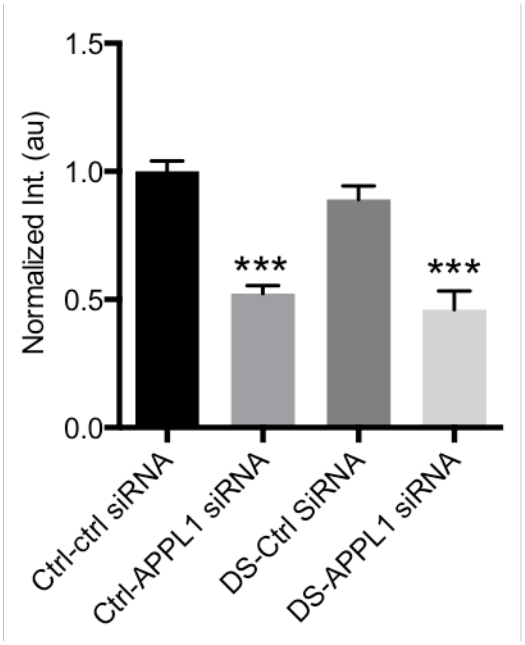

Supplementary Figure 7. APPL1 siRNA is sufficient to reduce APPL1 levels in DS fibroblasts. Both control and DS fibroblasts are transfected with control or APPL1 siRNA, and APPL1 levels are reduced about 50% in both cells (n=3 cultures, mean ± S.E.M., one-way ANOVA, Tukey's test, ***p<0.001)

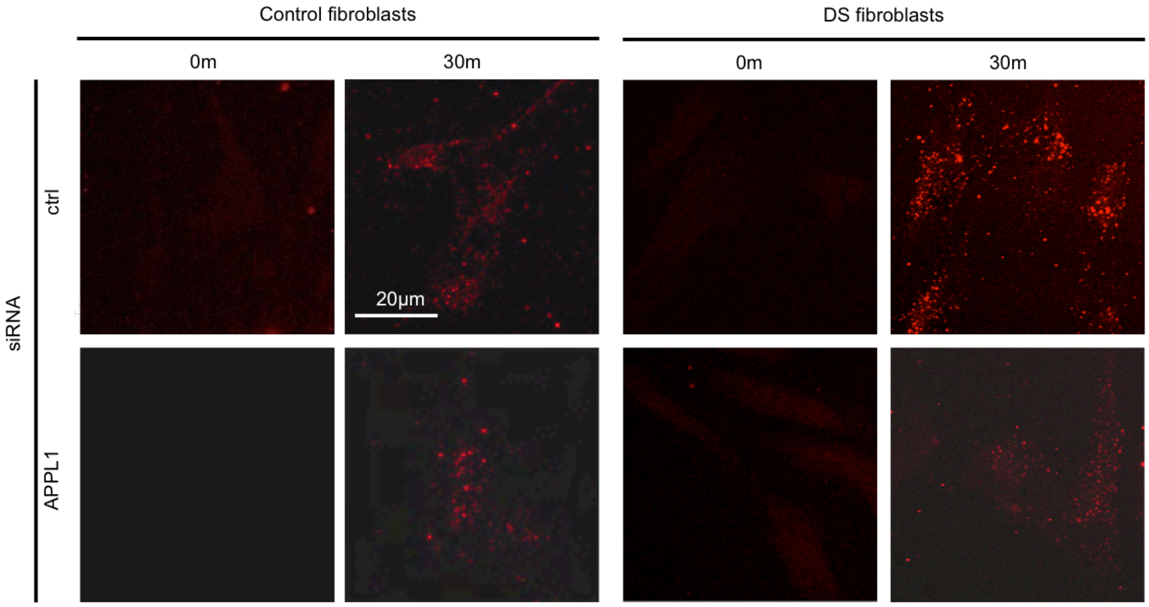

Supplementary Figure 8. Increased HRP uptake in DS fibroblasts can be decreased by reduction of APPL1 levels. Representative images of fluorescence of internalized HRP at 0 and 30 min after HRP addition to the medium in DS cells show significantly increased HRP uptake, consistent with accelerated endocytosis shown previously in DS fibroblasts measured by transferrin and EGF uptake[1, 2]. Increased HRP uptake is decreased by APPL1 siRNA treatment in DS fibroblasts while APPL1 siRNA has no effect on uptake in control cells. Quantitative analysis is shown in Fig. 5c.

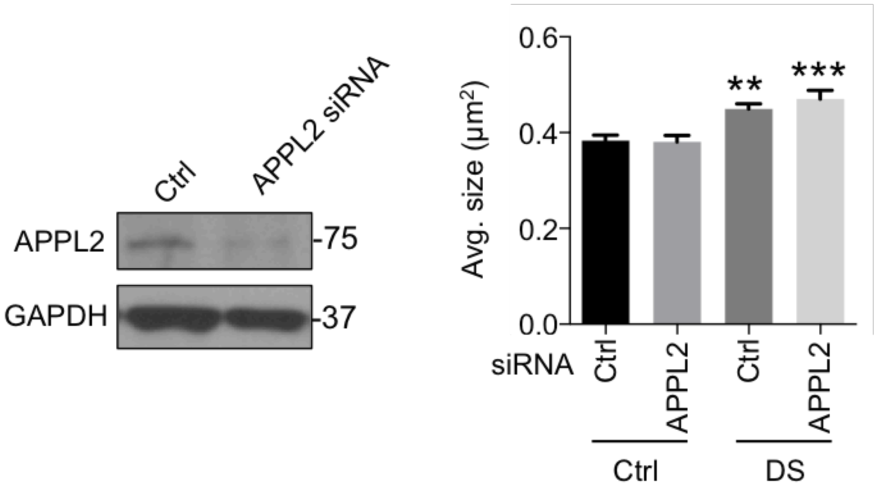

Supplementary Figure 9. APPL2 has no role in endosomal enlargement in DS fibroblasts. APPL2 siRNA significantly decreases APPL2 levels in DS cells as shown on representative immunoblots. GAPDH was used for loading control. Right-most panel showing quantified average size (cross-sectional area) of GFP-rab5 endosomes indicates no effect of APPL2 siRNA on endosomal enlargement in DS fibroblasts compared with DS fibroblasts treated with scrambled (ctrl) APPL2 siRNA. (n=30 cells, mean ± S.E.M., one-way ANOVA, Tukey's test, **p<0.01 and ***p<0.001).

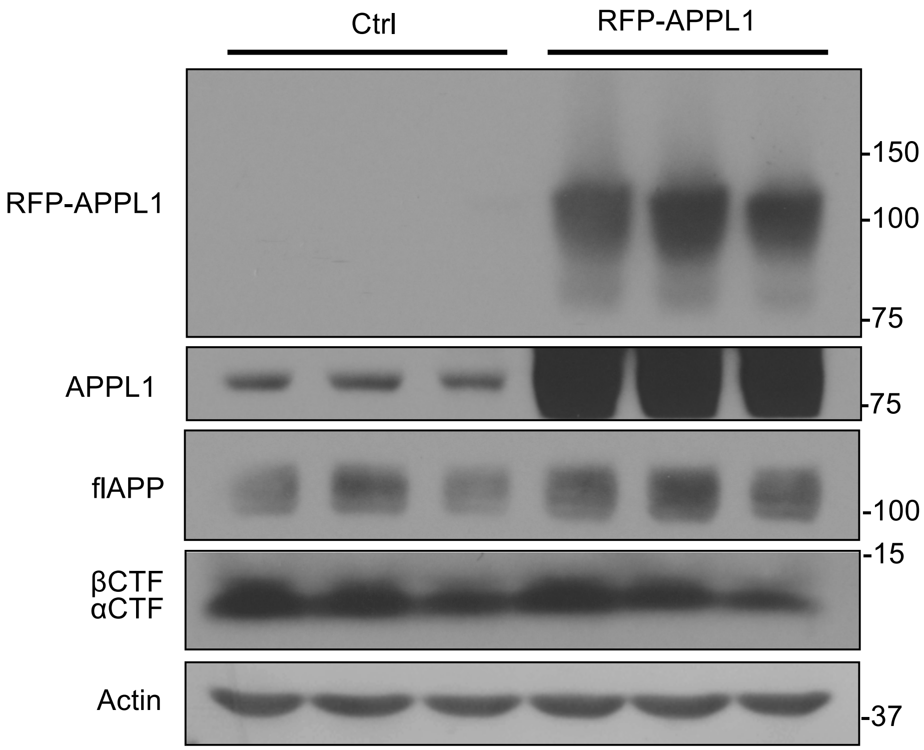

Supplementary Figure 10. RFP-APPL1 overexpression had no effect on βCTF generation. RFP-APPL1 overexpression in N2aAPP cells does not change βCTF levels (n=3 cultures).

Supplementary Table 1. Summary of endosomal analysis in primary neurons

	Morphology				Transport		
	**Number of endosomes**	**Average size (μm^2)**	**Density (n/axon)**	**Total area (μm^2/axon)**	**Number of endosomes**	**Average speed (μm/s)**	**Stationary endosomes (%)**
GFP-rhoB/RFP	134	0.33±0.02	6.7±0.4	2.21±0.15	110	1.96±0.29	17.9±4.2
GFP-rhoB/ rab5	133	0.42±0.02	6.7±0.3	2.78±0.26	107	0.57±0.11	22.7±4.0
GFP-rhoB/ APPwt	140	0.41±0.02	7.0±0.5	2.89±0.22	117	0.53±0.14	18.3±3.9
GFP-rab5/RFP	163	0.42±0.03	8.2±0.3	3.45±0.45	133	0.39±0.04	14.6±2.9
GFP-rab5/ APPwt	165	0.60±0.04	8.3±0.6	4.97±0.69	138	0.23±0.03	46.6±6.0
GFP-rab5/APPmv	152	0.42±0.03	7.6±0.7	3.17±0.43	132	0.45±0.04	18.6±4.7
GFP-rab5 +L685,458	199	0.61±0.03	9.9±0.7	6.07±0.47	190	0.23±0.02	41.6±3.5
GFP-rab5/ TFR	167	0.42±0.02	8.4±0.6	3.47±0.30	137	0.53±0.04	14.3±4.0
GFP-rab5 Q79L	117	0.94±0.07	5.9±0.6	5.52±0.53	102	0.15±0.02	65.2±7.7
GFP-rab5/ APPwt/ ctrl siRNA	141	0.56±0.04	7.1±0.5	3.93±0.27	116	0.15±0.02	53.0±6.8
GFP-rab5/ APPwt/ APPL1 siRNA	147	0.42±0.02	7.4±0.3	3.05±0.20	129	0.59±0.06	10.8±2.7
GFP-rab5/ APPwt/ APPL1wt	203	0.65±0.05	9.7±0.8	6.33±0.75	156	0.27±0.05	56.6±6.0
GFP-rab5/ APPmv/ APPL1wt	194	0.35±0.03	9.7±0.7	3.38±0.30	127	0.44±0.03	22.2±4.3
GFP-rab5/ APPwt/ APPL1ΔPTB	203	0.37±0.03	10.1±1.1	3.75±0.49	132	0.47±0.04	24.5±4.3

Supplementary Table 2. Analysis of mitochondrial transport.

	**Mito**	**Mito+APPwt**	**Mito+rab5**	**Mito+L685,458**
number of mito	70	67	68	68
Speed (μm/s)	0.24±0.07	0.24±0.07	0.25±0.08	0.23±0.05
Moving mito (%)	30.65±8.40	29.88±7.30	28.81±4.19	27.00±10.86

Supplementary Table 3. **Demographic data on the brains in this study**

	DX	AGE	PMI	Sex
1	Control	60	12.53	F
2	Control	61	10.08	M
3	Control	61	15.35	M
4	Control	63	17.67	M
5	Control	65	16.5	F
6	Control	65	18.07	M
7	Control	68	16.05	M
8	Control	69	8.08	F
9	Control	74	18.58	M
10	Control	75	12.1	F
11	Control	75	16.67	F
12	Control	79	15	F
13	Control	80	18.68	M
14	AD	62	4.3	F
15	AD	70	6.25	M
16	AD	71	11.87	M
17	AD	72	22.58	M
18	AD	72	10.17	M
19	AD	73	7.5	M
20	AD	73	11	M
21	AD	74	15	M
22	AD	74	10.33	M
23	AD	76	8.92	F
24	AD	76	6.3	M
25	AD	77	11.05	M
26	AD	79	13.67	M

Supplementary Information Materials and Methods

Cell culture and transfection. DS and control fibroblasts (Coriell Cell Repository, AG06922 and AG07095) were maintained and transfected with siRNA as described previously[2]. Cultured neurons, HEK293 cells, non-differentiated N2a cells, and fibroblasts were transfected by lipofectamine 2000 (Life Technologies) according to the manufacturer's protocol. A stable N2a cell line (ATCC, CCL-131) overexpressing APP (N2aAPP) was generated after linearized pcDNA3-APP695 plasmid (a gift from Dr. Paul Mathews) with PvuI was transfected into N2a cells. Transfected cells were plated on 35mm dishes for 2 days and subcultured into 100mm dishes at a 1:10 dilution. Cells were selected by incubating with G418 (0.6mg/ml, Life Technologies) for two weeks. Cells were used in each experiment from more than three independently prepared cultures.

DNA plasmids and siRNA. GFP-rab5 was cloned from HSV-myc-rab5 plasmid. The human rab5a gene was cut by SalI and BamHI and inserted into pEGFP-C1 vector (Clontech). Dominant active GFP-rab5 Q79L and dominant negative GFP-rab5 S34N were generated by PCR based site-directed mutagenesis (Stratagene). Plasmid coding transferrin receptor (TFR) was obtained from Addgene (12392). TFR was cut by XhoI and EcoRI and introduced into pRFP-monomer-C1 vector (Clontech). APPmv construct was generated from pcDNA-APP695 as described previously[3]. APPL1 was cloned from RFP-APPL1 obtained from Addgene (22202) by removing RFP tag. GFP-rhoB (pAcGFP1-Endo, Clontech) represented endogenous endosomes since it does not upregulate endocytosis when overexpressed[4]. To visualize mitochondria, dsRed-Mito (Clontech) was used. Mouse and human APPL1 siRNA, APPL2 siRNA, and control scramble siRNA were obtained from Life Technologies.

Immunoblots. Proteins were prepared from cells as described previously[5]. For membrane preparation, cleared lysates were separated by high-speed centrifugation (100,000g) for one hour at 4ºC. Pellet (P) was resuspended to equal volume of supernatant (S). Calnexin was used as a marker for membrane fraction and APP and βCTF were markers for APP overexpression. Proteins were analyzed from more than three independently prepared samples, and samples were duplicated. The intensity of bands was measured by Quantity One (BioRad). Primary antibodies were used at the following dilutions: 1:1000 for anti-rab5 (Abcam, ab18211), 1:5000 for anti-APPL1 (Abcam, ab59592), 1:200 for anti-APPL1 (Santa-Cruz, sc-55065), 1:1000 for C1/6.1 (Covance, SIG-39152), 1:1000 for 6E10 (Covance, SIG-39320), 1:5000 for anti-Calnexin (Santa Cruz, sc-11397), and 1:5000 for anti-GAPDH (Santa-Cruz, sc-32233).

Rab5 activity assay. The assay was performed according to the manufacturer's protocol (NewEast Biosciences, 83701). Transfected HEK293 cells were collected and lysed. Cell lysates were immunoprecipitated with an antibody against active (GTP-bound) rab5 (NewEast Biosciences, 26911), and immunoprecipitated samples were applied to immunoblots to measure active rab5 levels. 10% of total lysates was used as total rab5.

Co-immunoprecipitation. Co-IP experiment was performed using a co-IP kit (Pierce) following the manufacturer's protocol with samples from three independently prepared cultures. APPwt/APPL1wt, APPmv/APPL1wt or APPwt/APPL1ΔPTB plasmids were cotransfeced in HEK293 cells. Total lysates were pulled down with C1/6.1 or APPL1 antibody. APP_{AENATA}/APPL1wt or APPwt/APPL1wt were transfected in HEK293 cells, and lysates were immunoprecipitated with 6E10 or APPL1 antibody. To inhibit endocytosis, 20μM of MiTMAB™ (Abcam, ab120466)[6], a cell-permeable inhibitor for

dynamin I and II, were added in culture media of N2a cells overexpressing APPwt and APPL1wt for 12 hr before co-IP with C1/6.1 or APPL1 antibody. 20μM of Pro-Myristic Acid (Abcam, ab120476) was used as a negative control[6].

Time-lapse imaging. Analysis of fluorescently-labeled organelles was performed as described previously[7]. The sample size was determined according to the previous study[7]. Time-lapse images were acquired in a single focal plane at 5-second intervals for 50 images. Parameters of vesicle movement were generated using the kymograph plugin ImageJ. Traces of vesicle transport in kymographs were manually defined, and the average speed of vesicles was calculated by Δx (distance travelled, μm)/Δy (time, second) from each trace. Endosomes showing less than 0.2 μm/s in traces during recording were considered stationary.

FRAP Analysis. FRAP was carried out on a Zeiss LSM510 confocal microscope following the standard protocol described previously[8]. The sample size was based on the previous study[8] and the imageJ was used to analyze FRAP activity. GFP-rab5 was used for visualizing early endosomes.

HRP Uptake. HRP (Sigma, p6782) was diluted in DMEM medium for N2a cells or MEM medium for 2N/DS human fibroblasts to 0.5% was incubated for 0, 15, and 30 minutes. After incubation, cells grown on coverslips were washed, fixed with 4% paraformaldehyde (Electron Microscopy Sciences) for 20 minutes, washed again, blocked with blocking solution for 30 minutes, and incubated anti-HRP antibody conjugated with rhodamine (Jackson ImmunoResearch, 123-295-021, 1:500) for two hours. Coverslips were washed, mounted with Gelmount (Electron Microscopy Sciences), and imaged using a Zeiss LSM510 confocal microscope equipped with a 40X PlanApo oil immersion objective. The HRP intensity from each cell was determined by ImageJ and sample size was determined according to the previous study[2].

Abeta ELISA assay. ELISA measurements of secreted Aβ in culture media of N2aAPP transfected with APPL1 or APPL1 siRNA were performed as reported previously[2].

Preparation of AD brain tissue extracts. The cerebral cortex from human brains (Supplementary table 3) was homogenized in 2ml of a hypotonic buffer (20 mM Tris-HCl, pH 7.4, 1mM each EDTA, EGTA and DTT, 1mM β-glycerophosphate, 1mM NaF, 0.2mM $NaVaO_4$, 5g/ml each leupeptin, antipain and pepstatin and 1mM each of Benzamidine and PMSF). The homogenates were centrifuged at 15,000g for 30 min at 4°C. The resulting supernatant (S-1) was saved and the pellet was solubilized using a mixture of equal volume (300μl) of two buffers (1) 50mM Tris/HCl pH 7.4; 0.15M NaCl; 1% Sodium deoxycholate; 1% NP40, 2% SDS with a mixture of protease and phosphatase inhibitors) and (2) 8M Urea in 20mM Tris/HCl pH 7.4 then subjected to sonication. Protein content was determined by the BCA method. The supernatant and the sonicated pellet were used for western blot analysis. Brain extracts (supernatant and pellet) were analyzed for βCTFs using a Tris/Tricine PAGE system. Tris/Tricine gels (16.5% T, 3% C) were made from a 49.5% T, 5% C (separating gel) and a 49.5% T, 3.3% C (4% T, 3% C, stacking gel) and run on a vertical electrophoresis unit (Hoeffer Scientific Instruments), followed by immunoblot analysis. Antibody dilutions were used as follows: C1/6.1 (Covance, 1:1000) and 6E10 (Covance, 1:500). The immunoreactive bands were visualized with ECL reagent (Amersham) and the bands were quantified using MultiGauge (Fuji film) software. Full-length APP and its CTFs were measured and

quantified using C1/6.1. The identity of CTFs (C1-C5) was confirmed using 6E10. Based upon negative 6E10 immunoreactivity, band C5 was determined to be αCTF.

Immunolabeling and quantitative analysis. Immunocytochemistry and quantitative colocalization were carried out as described previously[1, 2, 5]. For GFP-rab5 endosome counting, small non-specific GFP-positive puncta ($<0.02\mu m^2$) were excluded from analysis. For human brain studies, 5μm paraffin sections obtained from prefrontal cortices of post-mortem AD brain (n=9) and neuropathologically normal controls (n=9) were used for immunolabeling. Primary antibodies were used at the following dilutions: 1:500 for anti-rab5 (Abcam) 1:50 for anti-rab5 (SantaCruz, sc-26566), 1:100 for anti-APPL1 (Abcam). Images were acquired using a 40X PlanApo oil immersion objective and a zoom of 4. Morphometric analysis of endosomes was performed using ImageJ as described previously[2]. For colocalization of APPL1 with rab5 in human brain, rab5-positive endosomes were first thresholded in ImageJ to create a binary mask using the Redirect Function. The corresponding APPL1 intensities were then measured in only the thresholded endosomes.

Preparation of nuclear and cytosolic fractions for p65 analysis. Cells were rinsed with ice-cold PBS and incubated with nucleus buffer (1mM K_2HPO_4/14mM $MgCl_2$/150mM NaCl/1 mM EGTA/0.1mM DTT/0.3% Triton X-100, pH 6.4) for 30 min at 4°C, then centrifuged at 1000g for 10 min in order to isolate nuclei (pellet) from cytosolic fraction (supernatant). Pellet was lysed in lysis buffer (36.25 mM Tris-HCl pH 6.8/50mM NaCl/1% SDS/5% glycerol/2.5% β-mercaptoethanol). Protein content was determined using Bradford protein assay (Bio-Rad, Hercules, CA, USA). Protein was transferred to nitrocellulose membrane. NF-kB p65 was detected with a rabbit monoclonal antibody (D14E12, Cell Signaling, xp-8242). Statistical analysis was performed using two-factor ANOVA with interaction.

References

1. Cataldo AM, Mathews PM, Boiteau AB, Hassinger LC, Peterhoff CM, Jiang Y *et al.* Down syndrome fibroblast model of Alzheimer-related endosome pathology: accelerated endocytosis promotes late endocytic defects. *Am J Pathol* 2008; **173**(2)**:** 370-384.

2. Jiang Y, Mullaney KA, Peterhoff CM, Che S, Schmidt SD, Boyer-Boiteau A *et al.* Alzheimer's-related endosome dysfunction in Down syndrome is A{beta}-independent but requires APP and is reversed by BACE-1 inhibition. *Proc Natl Acad Sci U S A* 2010; **107**(4)**:** 1630-1635.

3. Citron M, Teplow DB, Selkoe DJ. Generation of amyloid beta protein from its precursor is sequence specific. *Neuron* 1995; **14**(3)**:** 661-670.

4. Fernandez-Borja M, Janssen L, Verwoerd D, Hordijk P, Neefjes J. RhoB regulates endosome transport by promoting actin assembly on endosomal membranes through Dia1. *J Cell Sci* 2005; **118**(Pt 12)**:** 2661-2670.

5. Lee JH, Yu WH, Kumar A, Lee S, Mohan PS, Peterhoff CM *et al.* Lysosomal proteolysis and autophagy require presenilin 1 and are disrupted by Alzheimer-related PS1 mutations. *Cell* 2010; **141**(7)**:** 1146-1158.

6. Quan A, McGeachie AB, Keating DJ, van Dam EM, Rusak J, Chau N *et al.* Myristyl trimethyl ammonium bromide and octadecyl trimethyl ammonium bromide are surface-active small molecule dynamin inhibitors that block endocytosis mediated by dynamin I or dynamin II. *Mol Pharmacol* 2007; **72**(6)**:** 1425-1439.

7. Lee S, Sato Y, Nixon RA. Lysosomal Proteolysis Inhibition Selectively Disrupts Axonal Transport of Degradative Organelles and Causes an Alzheimer's-Like Axonal Dystrophy. *J Neurosci* 2011; **31**(21)**:** 7817-7830.

8. Brown HM, Van Epps HA, Goncharov A, Grant BD, Jin Y. The JIP3 scaffold protein UNC-16 regulates RAB-5 dependent membrane trafficking at C. elegans synapses. *Dev Neurobiol* 2009; **69**(2-3)**:** 174-190.
